# Identification and Comprehensive Prognostic Analysis of a Novel Chemokine-Related lncRNA Signature and Immune Landscape in Gastric Cancer

**DOI:** 10.3389/fcell.2021.797341

**Published:** 2022-01-14

**Authors:** Xiaolong Liang, Gangfeng Yu, Lang Zha, Xiong Guo, Anqi Cheng, Chuan Qin, Han Zhang, Ziwei Wang

**Affiliations:** ^1^ Department of Gastrointestinal Surgery, the First Affiliated Hospital of Chongqing Medical University, Chongqing, China; ^2^ Institute of Life Sciences, Chongqing Medical University, Chongqing, China; ^3^ Department of Digestive Oncology, Three Gorges Hospital, Chongqing University, Chongqing, China

**Keywords:** chemokine, immune, prognosis, gastric cancer, tumor mutation burden

## Abstract

Gastric cancer (GC) is a malignant tumor with poor survival outcomes. Immunotherapy can improve the prognosis of many cancers, including GC. However, in clinical practice, not all cancer patients are sensitive to immunotherapy. Therefore, it is essential to identify effective biomarkers for predicting the prognosis and immunotherapy sensitivity of GC. In recent years, chemokines have been widely reported to regulate the tumor microenvironment, especially the immune landscape. However, whether chemokine-related lncRNAs are associated with the prognosis and immune landscape of GC remains unclear. In this study, we first constructed a novel chemokine-related lncRNA risk model to predict the prognosis and immune landscape of GC patients. By using various algorithms, we identified 10 chemokine-related lncRNAs to construct the risk model. Then, we determined the prognostic efficiency and accuracy of the risk model. The effectiveness and accuracy of the risk model were further validated in the testing set and the entire set. In addition, our risk model exerted a crucial role in predicting the infiltration of immune cells, immune checkpoint genes expression, immunotherapy scores and tumor mutation burden of GC patients. In conclusion, our risk model has preferable prognostic performance and may provide crucial clues to formulate immunotherapy strategies for GC.

## Introduction

Gastric cancer (GC) is one of the most common malignant tumors worldwide, and its morbidity and mortality rank fifth and fourth, respectively (Sung et al., 2021). Although progress has been made in the diagnosis and treatment of GC, the overall survival time of patients has not improved significantly especially for advanced gastric cancer (AGC). The effect of immune checkpoint inhibitors has been previously investigated in patients with AGC. First-line treatment of PD-1/PD-L1 inhibitors could prolong OS and PFS of GC patients with CPS>10 or MSI-H (Shitara et al., 2020; Janjigian et al., 2021; Moehler et al., 2021). Results from a randomized phase III KEYNOTE-062 study indicated that AGC patients with a combined positive score (CPS) more than 10 or greater could benefit from first-line pembrolizumab (Smyth and Moehler, 2020). Huang et al. reviewed the efficacy and safety of third-line treatments for advanced gastric cancer (AGC). Among them, Nivolumab was one of the most effective third-line therapy drugs in prolonging overall survival (OS) of AGC especially for 1-year OS (Huang et al., 2021). These findings proved that PD-1/PD-L1 has an undeniable effect in patients with AGC. However, not all patients are sensitive to immunotherapy. Considering the poor outcome of GC and promising application use of immunotherapy, the identification of novel biomarkers for predicting prognosis and immune therapy response is helpful for disease stratification and developing GC treatment strategies.

Many biomarkers have been previously identified for predicting the prognosis of GC. Mismatch repair deficiency (MMRD) and microsatellite instability (MSI) were identified as positive prognostic biomarkers in GC patients treated with surgery alone and negative prognostic biomarkers in GC patients treated with chemotherapy (Smyth et al., 2017). Tumor mutation burden (TMB) was proved to be positively correlated with the disease-free survival (DFS) of microsatellite-stable (MSS) GC patients (Li et al., 2021). A combination of immune cell infiltration score and TMB score could be utilized to predict the survival of GC patients (Jiang et al., 2021a). Some noncoding RNAs such as circRNAs and lncRNAs could also act as diagnostic biomarkers or prognostic factors in GC (Yang et al., 2016; Shan et al., 2019). A recent study identified an immune-related signature composed of MAGED1, ACKR3, FZD2, and CTLA4 could be used to predict the prognosis of GC patients (Dai et al., 2021). These molecular analyses have increased our knowledge of GC biology and might provide new insights on GC therapy strategies.

Chemokines are a large class of cytokines with chemotactic activity. Chemokines were widely reported to regulate cancer progression and could be used as therapeutic targets (Mantovani et al., 2010). Dysregulation of chemokines and chemokine receptors were reported to be closely correlated with the progression of tumors including GC. For example, CC and CXC chemokines were reported to promote tumor angiogenesis, which is essential for tumor growth and metastasis (Santoni et al., 2014). CXCL5 chemokine could induce epithelial-mesenchymal transition (EMT) of GC cells thereby promoting GC metastasis (Mao et al., 2020). Another chemokine CXCL2 was reported to promote GC cell growth and peritoneal metastasis (Natsume et al., 2020). CXCL1 chemokine was reported to exert a tumor-promoting role through activating the VEGF pathway in GC (Wei et al., 2015). Chemokines and chemokine receptors also exert crucial roles in immunity and mainly affect the infiltration of various immune cells, thus affecting tumor progression. CCL2 chemokine and CCR2 chemokine receptor could regulate the infiltrating level of macrophages in hepatocellular carcinoma (Li et al., 2017). CCL2-CCR2 axis could affect the immune cell infiltration, which results in an induction of immune evasion in esophageal cancer (Yang et al., 2020). CCL28 chemokine could promote the infiltration of Treg cells, thereby promoting the progression of GC (Ji et al., 2020). CCL3 and CCL20 chemokines could recruit dendritic cell DCs, which could induce anti-tumor immunity of GC (He et al., 2010). In addition, some chemokines and chemokine receptors, such as CXCL8, CXCR4 and CXCL13, were proved to be promising prognostic biomarkers in GC (Jin K.et al., 2021; Pawluczuk et al., 2021; Xue et al., 2021). These findings indicated that chemokine-related genes exert crucial functions in tumors, especially in the tumor microenvironment.

LncRNAs are a subset of noncoding RNAs with a length of over 200 nucleotides that regulate the expression of many genes involved in cancer development (Chi et al., 2019; Fang et al., 2021; Yu et al., 2021). Apart from gene regulation, lncRNAs are also involved in regulating numerous biological processes involved in tumorigenesis (Bhan et al., 2017; Peng W.-X. et al., 2017; Fattahi et al., 2020). Mounting evidence indicated that lncRNAs have an undeniable prognosis prediction function in GC cancer. Prognostic signatures based on ferroptosis-related lncRNA, immune-related lncRNA and helicobacter pylori infection-related lncRNA were proved to have preferable prognosis prediction functions in GC (Ma et al., 2021; Pan J. et al., 2021; Wei et al., 2021; Xin et al., 2021). Apart from this, lncRNAs could be used for subtype identification and therapy response prediction of GC (Huang et al., 2021; Jiang et al., 2021b). LncRNAs have also been reported to modulate immunity (Yu et al., 2018). Various lncRNAs were identified as prognostic biomarkers and could be used to predict the immune landscape of multiple cancers (Hong et al., 2020; Shen et al., 2020; Xu et al., 2021).

At present, the role and type of immune landscape in the prognosis of gastric cancer remains largely unknown. Identification of infiltrating immune cells is associated with cancer prognosis and new immune therapeutic targets, which could provide meaningful clues for the future treatment of GC, especially for immunotherapy.

The correlation between chemokine-related lncRNAs and the immune landscape in GC has not been reported. In this study, we first constructed a multi-lncRNA risk model composed of 10 chemokine-related lncRNAs based on The Cancer Genome Atlas (TCGA) expression data. Then, we explored the prognostic efficiency and accuracy of the risk model. In addition, we explored the role of the risk model in predicting immune cell infiltration, immune checkpoint genes expression level and immunotherapy scores. Our results demonstrated that the lncRNA risk model shows preferable performance in predicting patient survival, immune cell infiltration and immunotherapy effectiveness.

## Results

### Identification of Chemokine-Related lncRNAs

**FIGURE 1:**
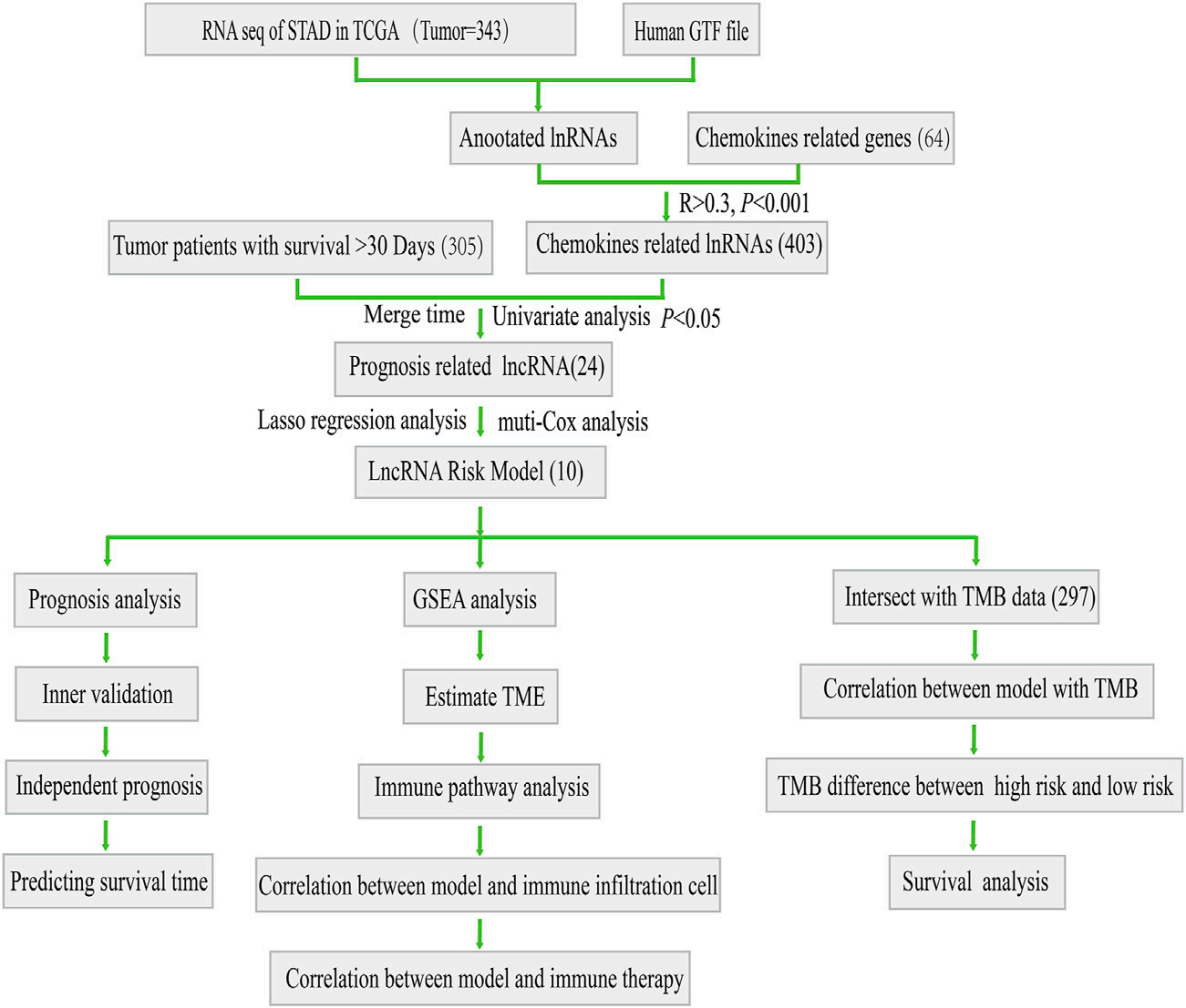
Flow chart of this study.

The workflow for this study was shown in Figure 1. First, we acquired the expression profiling data of 343 tumor samples and corresponding clinical information from The Cancer Genome Atlas (TCGA) database. We annotated the gene symbols to acquire the expression data of lncRNAs and mRNAs by using a human GTF file. Subsequently, we obtained 64 chemokine-related genes (Supplementary Table S2) (including chemokines and chemokine receptors) from four previous reviews concerned with chemokines or chemokine recptors (Zlotnik and Yoshie, 2012; Griffith et al., 2014; Sokol and Luster, 2015; Tiberio et al., 2018). Pearson’s correlation analysis (Pearson ratio > 0.3 and *p* < 0.001) was further conducted between these 64 chemokine-related genes and lncRNAs for screening chemokine-related lncRNAs. A total of 403 chemokine-related lncRNAs were identified and used for the subsequent analyses.

### Construction and Validation of the Risk Model

**FIGURE 2:**
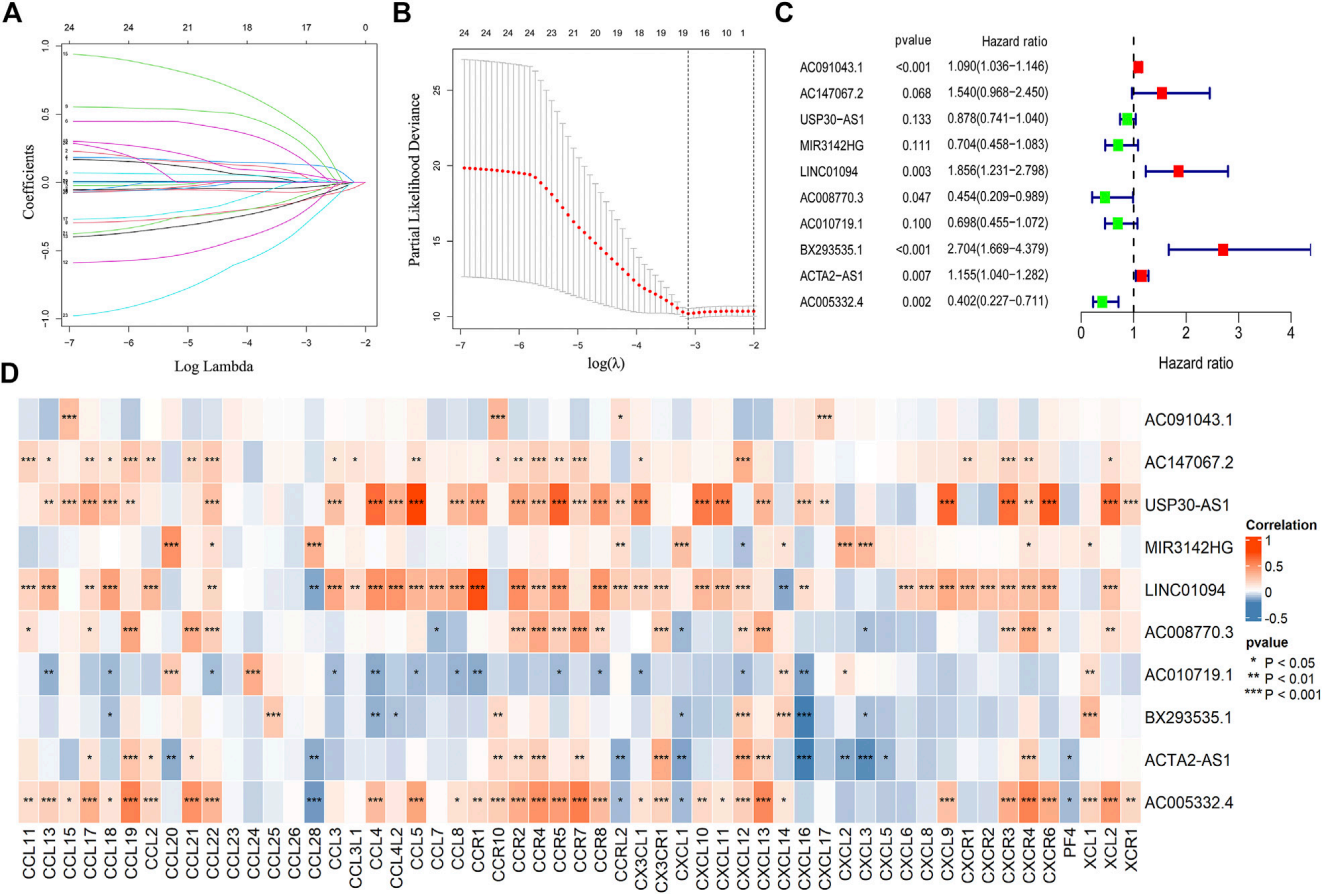
Construction of the risk model. The lasso regression analysis (lasso) was conducted for the construction of the risk model **(A,B)**. A total of 10 chemokine-related lncRNA were identified in the risk model **(C)**. The correlation between chemokines and lncRNAs in the risk model (*p < 0.05, **p < 0.01, ***p < 0.001) **(D)**.

After obtaining chemokine-related lncRNAs, we combined the survival status and survival time of gastric cancer (GC) patients with lncRNA expression data. We performed univariate analysis and obtained 24 prognostic chemokine-related lncRNAs. To further obtain the prognostic signature, we randomly divided 305 samples (the entire set) into two sets: training set (Supplementary Table S7) and testing set (Supplementary Table S8) at a ratio of 1:1. A total of 153 samples and 152 samples were enrolled in the training set and testing set, respectively. The training set was used for the establishment of the risk model. Then, lasso regression analysis was performed 1,000 times to recognize the potential survival-related combinations of the candidate chemokine-related lncRNAs in our study, which resulted in 17 optimal candidates (Figures 2A,B). To make our risk model more conducive to potential clinical application and cost-saving, we further conducted a multi-cox analysis on these 17 optimal candidates to reduce the number of lncRNAs in our model. Ten out of 17 lncRNAs were identified for the construction of the prognosis signature (Figure 2C and Supplementary Table S6). The coef value of each lncRNA was shown in Supplementary Table S5. The correlation between chemokines and 10 lncRNAs in the risk model was visualized by using a heatmap (Figure 2D). According to the median risk score, patients were divided into a high-risk group and a low-risk group. Principal component analysis (PCA) was used for dimensionality reduction of the entire genes, 403 chemokine-related genes and genes in the risk model according to the risk patterns of the risk model. Compared with the expression of all genes and 403 chemokine-related genes, only the risk model showed elevated efficiency in separating the high-risk and low-risk patients in all GC samples (Supplementary Figures S1E–G).

**FIGURE 3:**
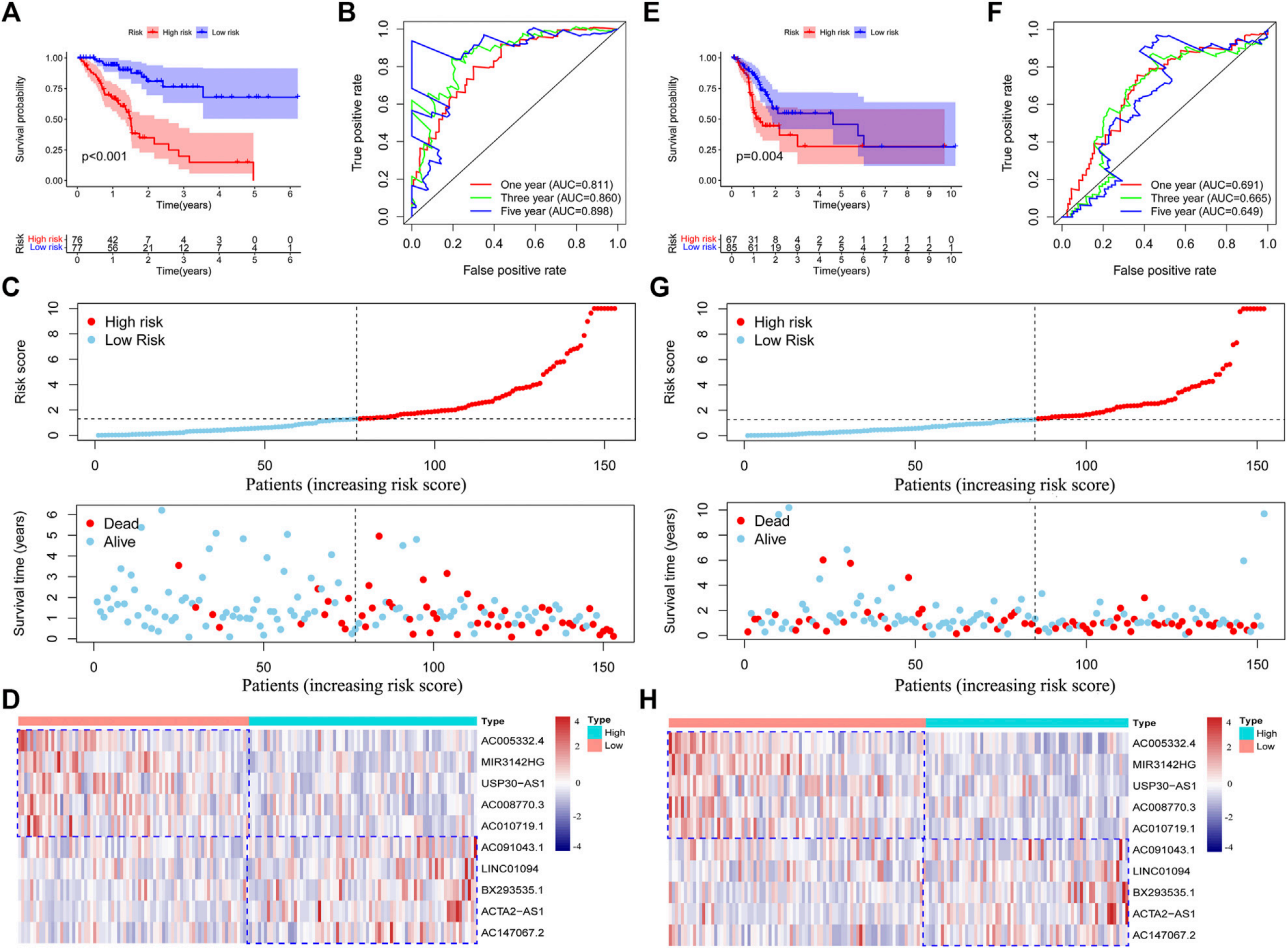
Validation of the risk model in the training set and testing set. Survival analysis of the training set and testing set **(A,E)**. Roc curves were plotted to assess the accuracy of the risk model in the training set and testing set **(B,F)**. The survival status of the patients in high-risk and low-risk groups **(C,G)**. The expression of the chemokine-related lncRNAs in the risk model was shown by using heatmap **(D,H)**. Low or high type represent the patients with low risk or high risk.

To further validate the efficiency of the risk model in predicting the survival of GC patients, we conducted survival analysis and found that low-risk group patients had a superior survival outcome than high-risk patients (Figure 3A). Next, we tested the accuracy of the risk model by using a time-dependent receiver operating characteristic (ROC) curve. The area under curve (AUC) value revealed that the risk model has enough efficiency in predicting the survival of GC patients (Figure 3B). In addition, we observed that there were more deaths in the high-risk group than in the low-risk group (Figure 3C). The expression of 10 lncRNAs in the risk model was visualized using a heatmap (Figure 3D).

### Inner Validation of Risk Model

To further validate the performance of the risk model, we conducted survival analysis in the testing set and the entire set. We observed that high-risk patients in the testing and entire set have poorer survival outcomes than low-risk patients (Figure 3E and Supplementary Figure S1A). Then, we tested the accuracy of the risk model in two sets by using a time-dependent ROC curve. As expected, we observed that our risk model has a preferable performance in both sets. The AUC values in the testing set and the entire set were 0.691 and 0.740 at one year (Figure 3F and Supplementary Figure S1B), respectively. After ranking the patients according to the median risk score, we found that the deaths incidence of low-risk patients in the training set and testing set were 11/77 (14.3%) and 28/85 (32.9%), respectively. However, the death rate of high-risk patients in the training set and testing set were 66/77 (85.7%) and 57/85 (67.1%), respectively (Figure 3G and Supplementary Figure S1C). We concluded that the high-risk group have more deaths than the low-risk group in both sets (*p* = 0.006). The expression of the 10 lncRNAs in two sets was shown in Figure 3H and Supplementary Figure S1D. These results indicated that our risk model has a good performance in predicting the survival outcome of GC patients.

Considering our risk model could not be validated in an external set, we obtained the expression data of colorectal cancer, the organ most closeted to stomach, (CRC) patients to validate the function of our risk model. We observed that our risk model has an undeniable value in predicting the survival time of CRC patients (Supplementary Figures S1H, I).

### Independent Prognostic Value of the Risk Model

**FIGURE 4:**
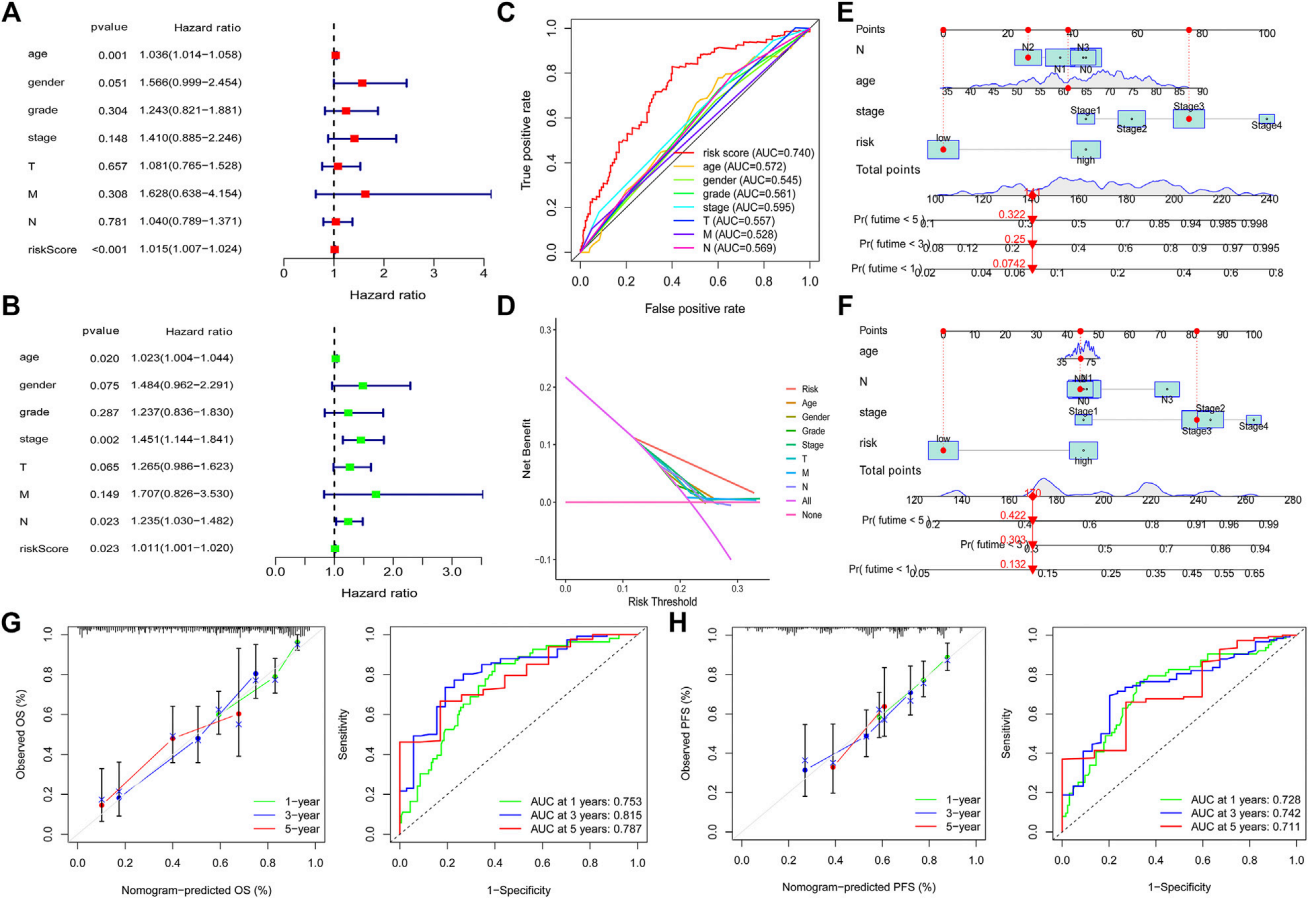
Independent prognosis value of the risk model. Univariate analysis and multivariable analysis were conducted to validate the independent prognosis value of the model **(A,B)**. Roc curves were performed to validate the superiority of the risk score in predicting patient’ survival **(C)**. Decision curve analysis (DCA) was conducted to confirm the superiority of the risk score in the clinical application **(D)**. Nomogram was plotted for the prediction of overall survival time **(E)** and progression-free survival time **(F)** in GC patients. The calibration curves and ROC curves were further plotted to determine the accuracy of the nomogram for OS and PFS at 1, 3 and 5 years, respectively **(G,H)**.

We also explored the correlation between the risk model and clinical characteristics of the GC patients. After excluding patients with unknown clinical features, no difference was observed in clinical characteristics between high-risk and low-risk patients (Table 1), which further validated the prognostic function of the risk model as not related to the clinical characteristics of the patients. To validate the independent prognostic value of the risk model, we conducted univariate analysis and multivariable analysis. We found that the risk score could be used as an independent prognostic index (Figures 4A,B). Then, we divided the patients into two groups according to different clinical characteristics and analyzed the survival outcome of the patients. Interestingly, we observed that low-risk group patients had better survival outcomes than high-risk group patients in all subgroups (Supplementary Figures S2A–G). In addition, we also divided 305 patients into a chemotherapy group (Supplementary Table S13) and a none-chemotherapy group (Supplementary Table S14). We tested the survival difference between low-risk and high-risk patients in two groups. Interestingly, we found that low-risk patients in the chemotherapy group have a better survival outcome than high-risk patients. However, there was no survival difference between low-risk and high-risk patients in the none-chemotherapy group (Supplementary Figures S3A–C). These findings indicated that the risk score could be used as an independent prognostic biomarker in all patients.

**TABLE 1:** Correlations between risk and clinicopathologic characteristics of GC patients.

Characteristic	Risk score			
	High (%) *n* = 127	Low (%) *n* = 145	χ2	*p*-value
Gender				
Male	75 (45.2)	91 (54.8)	0.39	*p* = 0.532
Female	52 (49.1)	54 (50.9)		
Age (Years)				
>65	63 (42.9)	84 (57.1)	1.889	*p* = 0.169
≤65	64 (51.2)	61 (48.8)		
Differentiation grade				
G1-G2	45 (45.0)	55 (55.0)	0.182	*p* = 0.670
G3	82 (47.7)	90 (52.3)		
Tumor size				
T1-T2	33 (45.8)	39 (54.2)	0.029	*p =* 0.865
T3-T4	94 (47.0)	106 (53.0)		
Metastasis				
M0	115 (45.1)	140 (54.9)	3.199	*p =* 0.074
M1	12 (70.5)	5 (29.5)		
Lymph node				
N0	37 (44.5)	46 (54.5)	0.214	*p =* 0.643
N1-N3	90 (50.3)	99 (49.7)		
Stages				
I-II	57 (44.9)	70 (54.1)	0.313	*p =* 0.576
III-IV	70 (48.3)	75 (51.7)		

To further validate that the risk model is superior to other clinical characteristics in prognostic predicting function, we conducted ROC curve analysis and decision curve analysis (DCA) at 1, 3 and 5 years, respectively. The results demonstrated that the risk model has an elevated efficiency compared with other clinical characteristics (Figures 4C,D and Supplementary Figures S3D–E). In addition, nomogram and calibration curves were plotted to determine the accuracy in predicting patients’ overall survival time (Figures 4E,G) and progression-free survival time (Figures 4F,H). The concordance index (C index) and ROC of the nomogram were also obtained to validate the accuracy of the nomogram. The value of C index is 0.739. As for the ROC of the OS nomogram, the AUC value of 1 year, 3 and 5 years were 0.753, 0.815 and 0.787, respectively (Figure 4G). We also observed that the predicted overall survival time and progression-free survival time were almost consistent with the actual survival time (Figures 4G,H), which further supports the risk model’s accuracy.

### Association Between the Risk Model and Immune-Related Pathways

**FIGURE 5:**
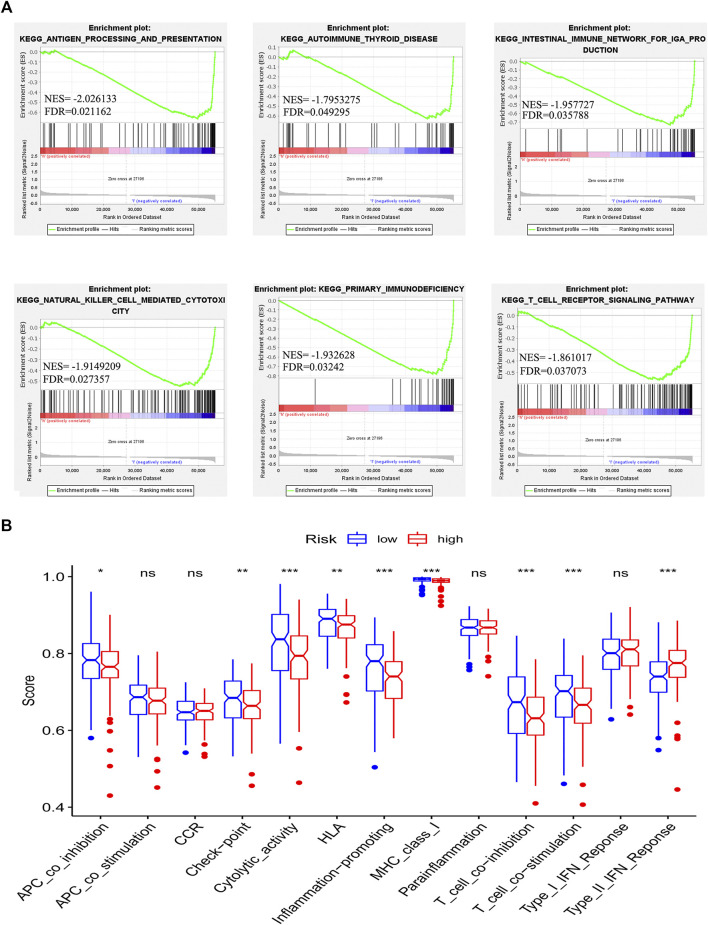
Correlation between immune microenvironment and risk model. Gene set enrichment analysis based on the chemokine-related lncRNAs risk model **(A)**. The difference in the enrichment of thirteen immune-related pathways between the low-risk group and the high-risk group was assessed (*p < 0.05, **p < 0.01, ***p < 0.001) **(B)**.

To detect the difference in KEGG enrichment between the low-risk and high-risk patients, we performed gene set enrichment analyses (GSEAs) and identified 21 enrichment pathways in the low-risk patients. Among these pathways, 6 out of 21 were immune-related pathways such as antigen processing and presentation, autoimmune thyroid disease, intestinal immune network, natural killer cell-mediated cytotoxicity, primary immunodeficiency and T cell receptor signaling pathway (Figure 5A). Subsequently, we determined the difference in 13 immune-related pathways between the high-risk group and low-risk group. Nine out of 13 pathways were identified to have a statistically significant difference between the two groups. Interestingly, among these nine immune-related pathways, eight pathways had a higher activity in the low-risk group, whereas one pathway showed a lower activity in the low-risk group (Figure 5B). This result was consistent with the enrichment of immune-related pathways in the low-risk group. These results indicated that the risk model is associated with the immune-related pathways in GC patients.

### Correlation Between the Risk Model and Immune Infiltration Cells

**FIGURE 6:**
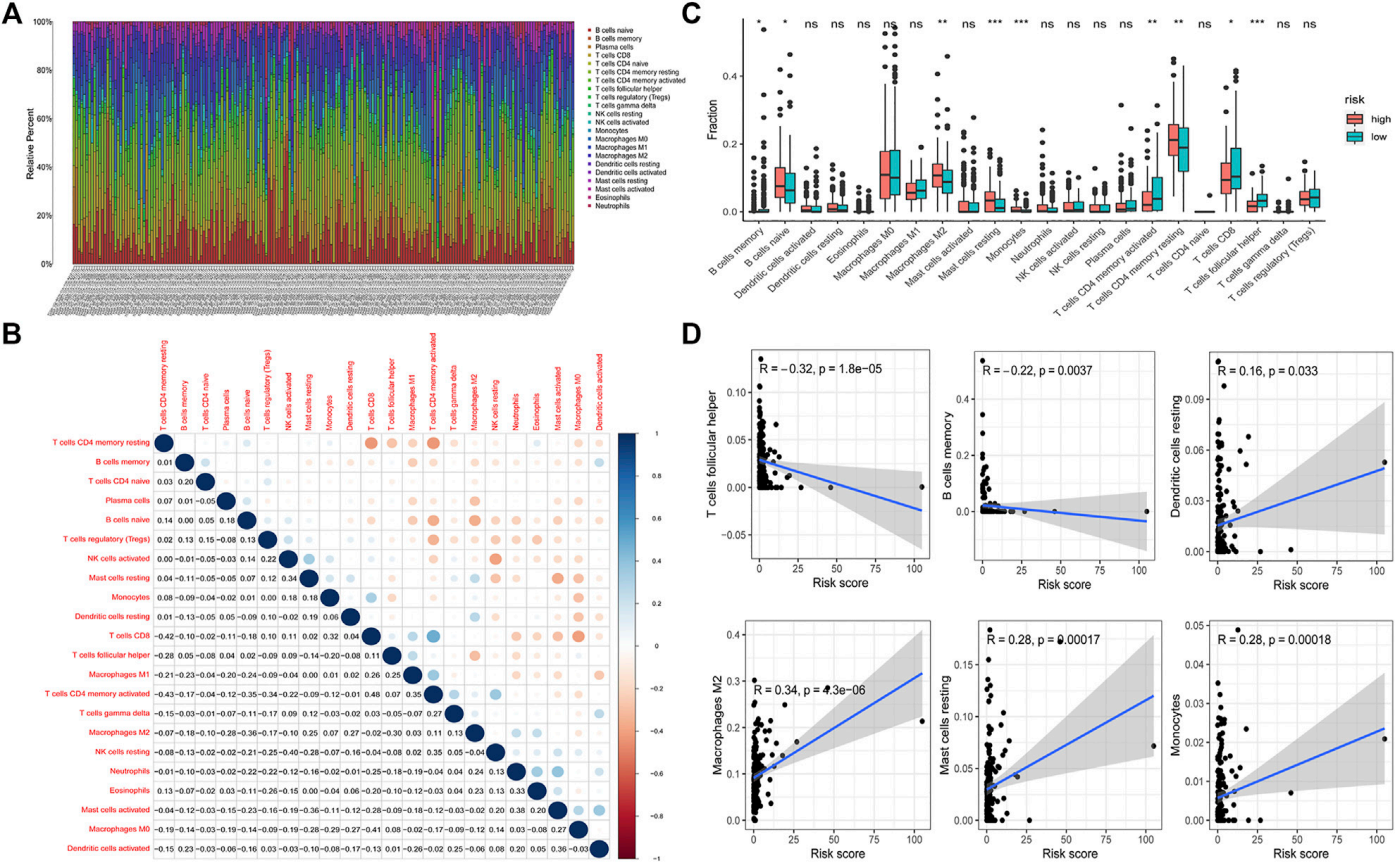
Tumor infiltrating immune cells in GC. CIBERSORT calculation method was used to calculate the infiltrating level of 22 tumor immune cells in GC patients **(A)**. Correlation between 22 tumor infiltrating immune cells was visualized. The red plot represents the negative correlation between two immune cells, while the blue plot represents the positive correlation between two immune cells. The larger the shape of the point, the stronger the correlation **(B)**. The difference of the tumor infiltrating immune cells between the low-risk group and high-risk group (*p < 0.05, **p < 0.01, ***p < 0.001) **(C)**. Correlation between the risk score and infiltrating level of T cells follicular helper, B cells memory, dendritic cells resting, macrophages M2, mast cells resting and monocytes **(D)**.

Based on the above results, we speculated that the low-risk group and the high-risk group have different immune microenvironment statuses. To validate our hypothesis, the infiltration status was calculated by using the CIBERSORT analysis. The infiltration of 22 immune cells was visualized by using a bar plot graph (Figure 6A). We visualized the infiltration of 22 immune cells in groups according to the risk pattern. Results demonstrated that the infiltration pattern of 22 immune cells in low-risk group patients is obviously different from that in high-risk group (Supplementary Figures S3F, G). Then, we determined the correlation among 22 immune cells. The results demonstrated that most types of T cells have a negative correlation with macrophages, mast cells and dendritic cells (Figure 6B). In addition, we determined the difference in immune infiltration cells between the low-risk group and high-risk group. We observed that low-risk group patients had a higher infiltration of memory B cells, activated memory CD4 T cells, CD8 T cells and follicular helper T cells. However, a higher infiltration of naive B cells, M2 macrophages, resting mast cells, monocytes and resting memory T cells was detected in the high-risk group (Figure 6C). Furthermore, we detected the correlation between the infiltration of immune cells and the risk score. We observed that follicular helper T cells and memory B cells have a negative correlation with the risk score, which indicated that patients with lower risk scores have higher infiltration of these two immune cells (Figure 6D). In contrast, resting dendritic cells, M2 macrophages, resting mast cells and monocytes had a positive correlation with the risk score, which indicated that patients with higher risk score have more infiltration of these immune cells (Figure 6D). Our results suggested that the risk model could be used to predict the infiltration of immune cells.

### Clinical Value of the Risk Model in Immunotherapy

**FIGURE 7:**
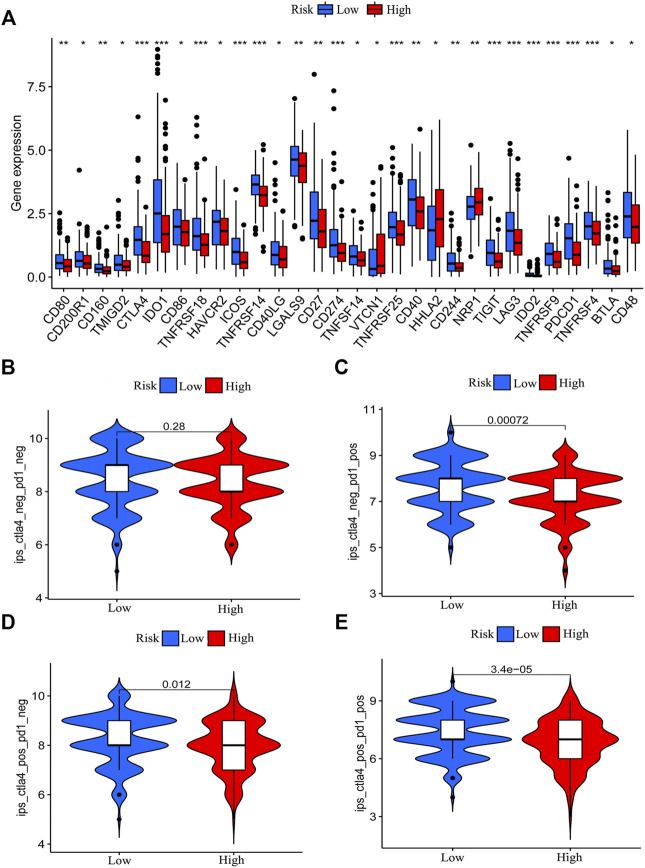
Correlation between risk model and immune checkpoint genes and immunotherapy score. The difference in the expression of immune checkpoint genes between the low-risk group and the high-risk group was determined (*p < 0.05, **p < 0.01, ***p < 0.001) **(A)**. The immunotherapy scores of patients with positive status of CTLA4 or PD-1 in the low-risk group patients are higher than that of high-risk group patients **(B–E)**.

The expression of immune checkpoint genes was reported to be associated with immunotherapy efficiency (Burugu et al., 2018). Patients with higher expression of PD-L1 have better immunotherapy outcomes in NSCLC (Sharma et al., 2021). In addition, we found that patients with PD-L1 combine positive score (CPS) > 10 could benefit more from PD-1 or PD-L1 immunotherapy (Shitara et al., 2020; Janjigian et al., 2021; Moehler et al., 2021). Elizabeth C et al. also analyzed the results of KEYNOTE-062 and found that AGC patients with PD-L1 combined positive score (CPS) more than 10 or greater could benefit more from pembrolizumab than patients with a CPS of 1 or greater (Smyth and Moehler, 2020). To evaluate the expression difference of immune checkpoint genes, we compared the expression of immune checkpoint genes between the low-risk and high-risk patients. The results demonstrated that the low-risk patients showed elevated expression of most immune checkpoint genes (Figure 7A), which indicates that low-risk patients might be more sensitive to immunotherapy although clinical evidence should be required. To validate our results, we obtained the immunotherapy score data from TCIA (https://tcia.at/) and compared the difference in immunotherapy score between the two groups. Immunotherapy score was derived in an unbiased manner using machine learning by considering the four major categories of genes that determine immunogenicity (effector cells, immunosuppressive cells, MHC molecules, and immunomodulators) by the gene expression of the cell types these comprise (e.g., activated CD4^+^ T cells, activated CD8^+^ T cells). The immunotherapy score is positively correlated with immunogenicity. Results demonstrated that the low-risk group patients with single positivity for CTLA4 or PD-1 and double positivity for CTLA4 and PD-1 had higher immunotherapy scores (Figures 7B–E). We also utilized Tumor Immune Dysfunction and Exclusion (TIDE) score to prove immune response difference between the high-risk group and low-risk group. Result demonstrated that low-risk group has a relative lower TIDE prediction score, which indicated a potential better immune therapy response in low-risk group (Supplementary Figure S3J).

### Correlation Between the Risk Model and Tumor Mutation Burden

**FIGURE 8:**
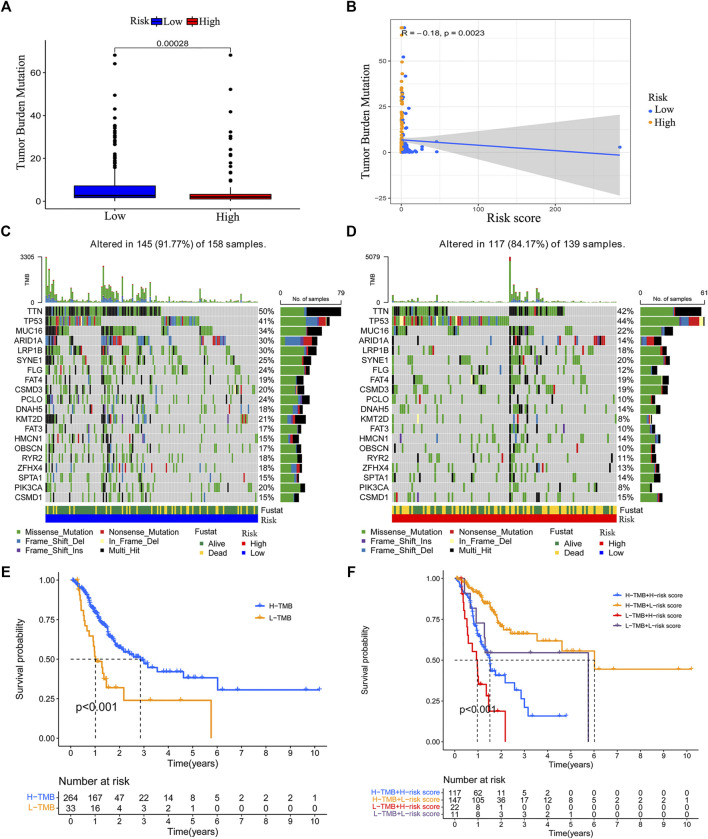
Correlation between the risk model and tumor mutation burden (TMB). Boxplot was used to visualize the TMB level between the low-risk group and high-risk group **(A)**. The risk score is negatively correlated with the TMB level **(B)**. The top 20 genes’ TMB in the low-risk group and high-risk group **(C–D)**. The survival difference between the high TMB group and low TMB group **(E)**. The survival status of patients with low or high risk-score in the high TMB group and low TMB group **(F)**.

By using “maftools” of R, we acquired the TMB data of GC. We compared the TMB difference between the low-risk group and high-risk group. We found that low-risk group patients had a higher TMB level (Figure 8A). The risk score is negatively correlated with TMB level (Figure 8B). We also analyzed the TMB status in the low-risk and high-risk groups. Except for the mutation of TP53, the mutation of other genes was higher in the low-risk group (Figures 8C,D). We grouped the patients according to the TMB level and analyzed the survival outcomes. We found that patients with higher TMB had better outcomes (Figure 8E). In addition, high TMB patients with lower risk scores had the best survival outcome. However, low TMB patients with higher risk scores have the worst survival outcome (Figure 8F).

### Expression Validation of Ten lncRNAs in Our Risk Model

**FIGURE 9:**
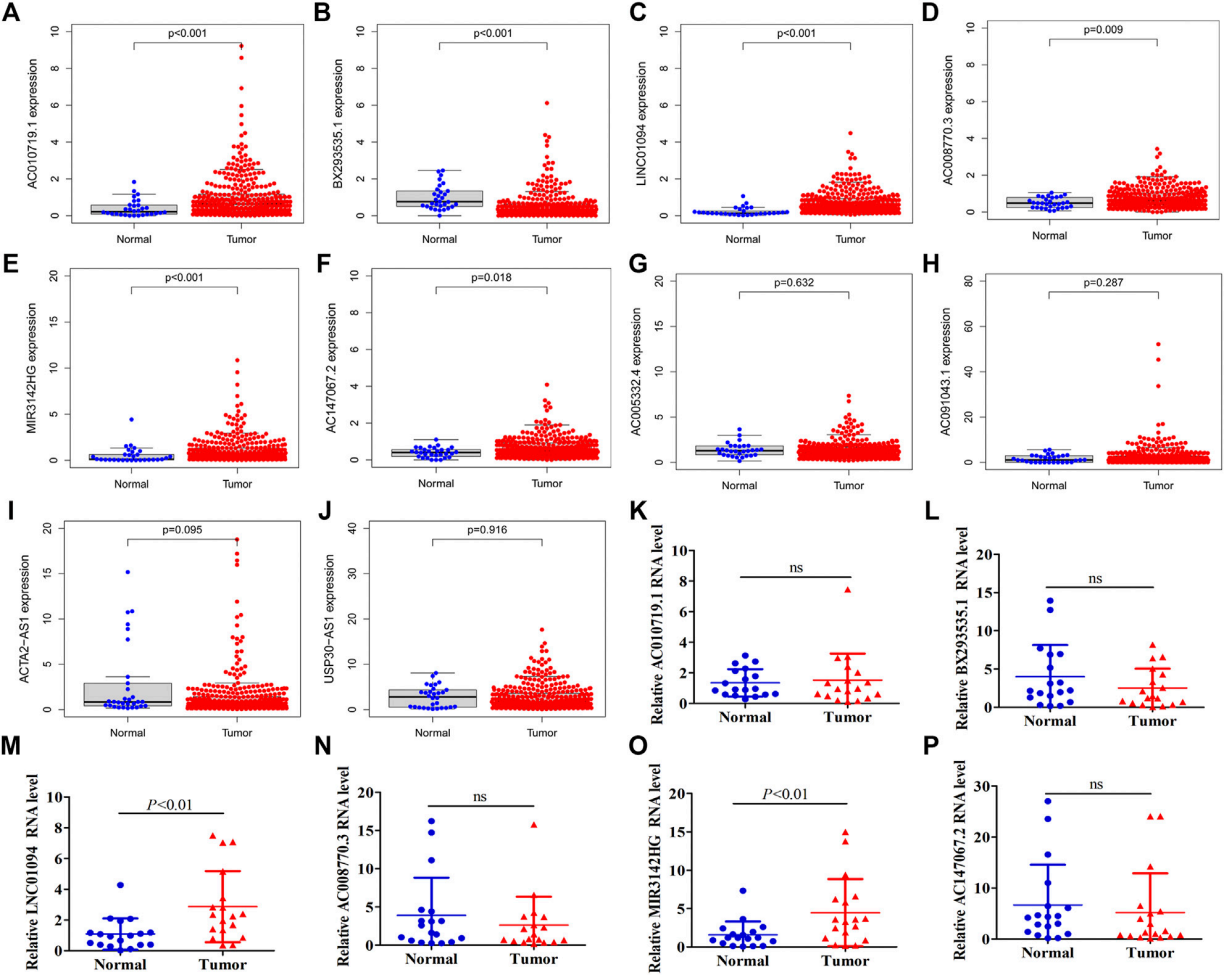
Expression validation of ten lncRNAs in our risk model. A total of six lncRNAs were identified to be differentially expressed between normal tissues and tumor tissue of the TCGA dataset **(A–J)**. Expression of AC010719.1, BX293535.1, MIR3142HG, LINC01094, AC008770.3 and AC147067.2 between tumor tissues and adjacent normal tissues in 18 clinic samples **(K–P)**.

The above results indicated that ten chemokine-related lncRNAs of the risk model (AC010719.1, BX293535.1, LINC01094, AC008770.3, MIR3142HG, AC147067.2, AC005332.4, AC091043.1, ACTA2-AS1, USP30-AS1) were associated with patients’ survival and immune landscape. To find the most valuable lncRNAs in our risk model, we determined the expression of each lncRNA in TCGA coherent. Six out of ten lncRNAs (AC010719.1, BX293535.1, LINC01094, AC008770.3, MIR3142HG and AC147067.2) were differentially expressed between tumor tissues and normal tissues (Figures 9A–J). Compared with normal tissues, only BX293535.1 exhibited a lower expression level in tumor tissues (Figure 9B). In addition, we collected 18 paired GC samples and performed qRT-PCR to validate the differences of these six lncRNAs in clinic samples. Interestingly, we observed that only LINC01094 and MIR3142HG were differentially expressed between tumor tissues and paired adjacent normal tissues (Figure 9K–P). These results indicated that LINC01094 and MIR3142HG might exert a more crucial function in GC development.

## Discussion

Traditionally, patients diagnosed with GC are treated with sequential chemotherapy such as the combination of platinum and fluoropyrimidine (Smyth et al., 2020). However, the overall survival of patients is still very low (Spolverato et al., 2014), especially the median survival time of advanced gastric cancer (AGC) is less than one year (Smyth et al., 2020). Immunotherapy can prolong the overall survival of many cancer patients (Herbst et al., 2016; McGranahan et al., 2016; Jin T. et al., 2021). However, immunotherapy is not effective for all patients. The effectiveness of immunotherapy is associated with many factors, such as the infiltration state of immune cells (Anfray et al., 2019; Zhang et al., 2019), the expression level of immune checkpoint genes (Burugu et al., 2018) and the state of somatic mutations (Allgäuer et al., 2018; Zhao et al., 2019). Therefore, it is very important to find effective biomarkers that could be used to predict patient prognosis and immunotherapy sensitivity.

Chemokines are a large class of cytokines with chemotactic activity. It has been reported that chemokines exert crucial functions in the tumor microenvironment, especially in the immune microenvironment (Bian et al., 2019; Xun et al., 2020). Different types of immune cells could be recruited into the tumor microenvironment via interactions between chemokines and chemokine receptors (Nagarsheth et al., 2017). Altered expression levels of chemokines in malignant tumors are associated with angiogenesis, proliferation, metastasis and recruitment and activation of immune cells in multiple tumors (Strieter et al., 2005; Teicher and Fricker, 2010; Santoni et al., 2014; Li et al., 2017; Yang et al., 2017; Liang et al., 2018; Mo et al., 2020; Yang et al., 2020). Many anti-chemokine drugs have been used in combination with other antitumor drugs in cancer treatment (Feig et al., 2013; Salazar et al., 2018). Therefore, it is essential to explore the function of chemokines and their related genes.

In this study, we first identified a novel chemokine-related lncRNA prognostic signature based on the expression data of patients in the TCGA database. In brief, we acquired the RNA sequence profiles of 343 tumor samples from the TCGA database. By using the human GTF file, we annotated the mRNA and lncRNA from the RNA sequence results. To obtain chemokine-related lncRNAs, we conducted co-expression analysis between certified chemokine-related genes and lncRNAs (Zlotnik and Yoshie, 2012; Griffith et al., 2014; Sokol and Luster, 2015; Tiberio et al., 2018). A total of 403 chemokine-related lncRNAs were identified and used for univariate analysis to obtain prognostic chemokine-related lncRNAs. Then, lasso regression analysis (LASSO) and multi-cox analysis methods were performed to screen chemokine-related lncRNAs for the construction of the model. Ten chemokine-related lncRNAs (AC010719.1, BX293535.1, LINC01094, AC008770.3, MIR3142HG, AC147067.2, AC005332.4, AC091043.1, ACTA2-AS1, USP30-AS1) were identified in the risk model. Among these lncRNAs, AC091043.1, USP30-AS1, MIR3142HG, LINC01094 and ACTA2-AS1 were reported to regulate the progression of various tumors, while others were reported for the first time (Hadjicharalambous et al., 2018; Xu et al., 2020; Pan et al., 2021a; Wan et al., 2021). After obtaining the risk model, we divided patients into a high-risk group and a low-risk group according to the median risk score. Then, Principal component analysis (PCA) was performed for dimensionality reduction and model identification of the entire gene expression profile, 403 chemokine-related genes and a risk model (Li et al., 2020). As expected, we found that only the risk model showed elevated efficiency in separating the high-risk patients and low-risk patients, which further validates the efficiency of the risk model in separating high-risk patients and low-risk patients.

To explore the function of the risk model in predicting patients’ survival outcomes. We performed survival analysis and found that low-risk group patients have better survival outcomes than high-risk group patients in the training set. The area under curve (AUC) values of the ROC curve at one, three and five years exceeded 0.8, which confirmed the accuracy of our risk model. The efficiency and accuracy of the risk model in the testing set and entire set were also determined. In addition, we explored the independent prognostic function of the risk model. The results demonstrated that the risk score could be used as an independent prognostic biomarker in GC. All patients with different clinical characteristics in the low-risk group had better survival outcomes. ROC curve and decision curve analysis (DCA) were conducted to validate the accuracy of the risk score in an independent prognostic function. In addition, the predicted survival time from the nomogram was almost consistent with the actual survival time. These results indicated that our risk model has enough efficiency in predicting the prognosis of GC patients.

The immune system plays a crucial role in the development of cancer. Chemokines play crucial functions in the tumor microenvironment, especially in the immune microenvironment (Bian et al., 2019; Xun et al., 2020). To explore whether the chemokine-related lncRNA risk model also functions in the immune microenvironment, we performed gene set enrichment analyses (GSEA) and identified six immune-related pathways, antigen processing and presentation, autoimmune thyroid disease, intestinal immune network, natural killer cell-mediated cytotoxicity, primary immune deficiency and T cell receptor signaling pathway, that were enriched in the low-risk group (Tang et al., 2021). In addition, we observed that patients in the low-risk group showed an elevated score in immune-related pathways. Therefore, we speculated that the risk model may regulate immunotherapy by affecting the immune infiltration cells of GC. The status of immune infiltration cells was also reported to be associated with the response to immunotherapy (Anfray et al., 2019; Zhang et al., 2019). Then, we analyzed the proportion of infiltrating immune cells in GC tissue. We observed that the low-risk group had a higher infiltration of memory B cells, activated memory CD4 T cells, CD8 T cells and follicular helper T cells. However, the high-risk group had a higher infiltration of naive B cells, M2 macrophages, resting mast cells, monocytes and resting memory T cells. High infiltration of helper T cells, memory CD4 T cells and CD8^+^ T cells is reported to be associated with better survival outcomes in patients with cancers (Melssen and SlingluffJr, 2017; Kim H. S. et al., 2021). Patients with more CD4^+^ and CD8^+^ T cell infiltration experienced a superior treatment response from immunotherapy than those with less infiltration (Zander et al., 2019; Niogret et al., 2021; Pan et al., 2021b). In contrast, M2 macrophages, resting mast cells and monocytes exert tumor-promoting functions. Monocytes can affect the tumor microenvironment through various mechanisms that induce angiogenesis, immune tolerance, and increased dissemination of tumor cells (Ugel et al., 2021). Infiltration of monocytes is associated with cancer progression, including GC (Peng L.-s. et al., 2017; Wang et al., 2017; Cavassani et al., 2018). Mast cells play a tumor-promoting role in gastric cancer by releasing angiogenic factors and lymphangiogenic factors (Sammarco et al., 2019). Macrophages in solid tumors are associated with poor prognosis and might enhance tumor progression and metastasis (Qian and Pollard, 2010; Cassetta and Pollard, 2018). M2 macrophages are related to the EMT and progression of GC (Guan et al., 2021). These results support the use of our risk model as a biomarker for predicting the GC immune landscape.

Immune checkpoint genes’ expression level and tumor mutation burden (TMB) are effective indicators for immunotherapy. Patients with higher expression of immune checkpoint genes and higher somatic mutations might have better immunotherapy effectiveness (Allgäuer et al., 2018; Burugu et al., 2018; Zhao et al., 2019). To further understand the function of the risk model in the immune landscape, we analyzed the expression of immune factors and found that patients in the low-risk group had a relatively higher expression of various checkpoint genes. In addition, we compared the TMB status between the low-risk and high-risk patients. The risk score obtained from the risk model is negatively correlated with TMB. Patients with lower risk scores have a higher level of TMB. Thus, we speculated that low-risk patients might be more sensitive to immunotherapy. Based on this hypothesis, we downloaded the immunotherapy score data of GC and assessed the sensitivity of high-risk and low-risk group patients to immunotherapy. We found that low-risk patients with single positivity for CTLA4 or PD-1 and double positivity for CTLA4+PD-1 had higher immunotherapy scores. The survival analysis concerned with TMB revealed that high TMB patients with lower risk scores had the best survival outcome, and low TMB patients with higher risk scores had the worst survival outcome. According to the above results, we concluded that the chemokine-related lncRNA risk model could be used to predict the immunotherapy sensitivity of GC.

Recently, many studies constructed prognostic signatures in GC. All these studies aim to find a reliable signature for predicting prognosis, immune cells infiltration and immune response of GC. Dai identified that low-risk patients in their risk model have a higher tumor mutation burden (TMB) score and immunotherapy score than that in high-risk group (Dai et al., 2021), which is similar to our results. Ma established an immune-related lncRNA signature which has a preferable performance in prognosis and immune cell infiltration prediction. They observed that high-risk patients have a relatively higher infiltration of M2 macrophages and T cells regulatory (Ma et al., 2021). In our study, risk score was revealed to be positively correlated with the infiltration of M2 macrophages. Unexpectedly, there was no obvious difference in the infiltration of T cells regulatory between the two groups. Unlike other studies, Kim et al. constructed a novel tumor immune microenvironment (TIM) classification system. They found that TIM of GC could be influenced by frameshift mutations and tumor mutational burden (Kim H. et al., 2021). In our study, we only observed that risk score is closely correlated with immune cells infiltration and TMB. However, whether the infiltration of immune cells could be affected by TMB needs further research.

Despite our positive findings, we recognized that our study has some limitations. We obtained a risk model that could be used to predict patients’ survival outcomes and immune landscape. We didn’t perform independent validation of the risk model, which might lead to a risk of overfitting the model. In this regard, we performed 1,000 times lasso regression analysis. After obtaining the risk model from the training set, we validate the prognostic function of the model in the testing set and entire set. We also validated that our risk model has a good performance in predicting the survival time of CRC patients. These results indicated our model is reliable in predicting the prognosis of gastrointestinal cancer.

Additionally, these ten lncRNAs have not been previously reported to be associated with GC except LINC01094. LINC01094 was used for the construction of another signature to predict the prognosis of GC patients (Zhang et al., 2021). To find the most valuable lncRNAs in our risk model, we determined the expression of ten lncRNAs in the TCGA dataset and 18 clinic samples. Two lncRNAs (LINC01094 and MIR3142HG) were identified to be differentially expressed between normal tissues and tumor tissues both in the TCGA dataset and 18 clinic samples. These results indicated that these two lncRNAs (LINC01094 and MIR3142HG) in the risk model might exert vital function in the prognosis and immune infiltration of GC patients. We will explore the association between these two lncRNAs and GC in further study.

In conclusion, we constructed a chemokine-related lncRNA risk model in GC. The risk model could be used to predict the prognosis of GC patients. The risk model also exerts a crucial function in predicting the immune landscape of GC patients. These results could provide insights for prognosis prediction of GC patients and might provide valuable clues for immunotherapy in GC.

## Materials and Methods

### Data Acquisition and Processing

The RNA sequence data of gastric cancer (GC) and colorectal cancer (CRC) patients and their corresponding clinical information (Supplementary Table S1) were obtained from The Cancer Genome Atlas (TCGA) (https://tcga-data.nci.nih.gov/tcga/). Patients with survived time more than 30 days were enrolled. The human GTF file download from Ensembl (http://asia.ensembl.org) was used to acquire mRNA and lncRNA expression data from transcriptome data.

### Acquiring of the Prognostic Chemokine-Related lncRNAs

According to four previous reviews concerned with chemokines or chemokine receptors (Zlotnik and Yoshie, 2012; Griffith et al., 2014; Sokol and Luster, 2015; Tiberio et al., 2018), we obtained 64 chemokine genes (Supplementary Table S2). Then, the expression of these 64 chemokine genes was extracted from the mRNA matrix of TCGA STAD by using the “limma” package of R software. Based on these 64 chemokines, we screened chemokine-related lncRNAs from lncRNA matrix by using Pearson’s correlation analysis (Pearson ratio >0.3 and *p* < 0.001), and 403 chemokine-related lncRNAs were identified (Supplementary Table S3). Subsequently, univariate analysis was performed to determine prognosis-related lncRNAs. A total of 24 prognostic chemokine-related lncRNAs were identified (Supplementary Table S4).

### Establishment of the Risk Model

The training set was used to construct the risk model, and the entire set (Supplementary Table S6) and testing set were used for the validation of the risk model. In brief, the lasso regression analysis and multi-cox analysis were utilized to construct the lncRNA risk model by using 24 prognostic chemokine-related lncRNAs. We identified 10 chemokine-related lncRNAs (Supplementary Table S5) to establish the risk model. The calculation formula of the risk score is as follows:

$$Risk\;score\left(patients\right)=\overset{n}{\underset{i=1}{\sum }}coefficient\left(gene\;i\right)*expression\left(gene\;i\right)$$

In this formula, n, i, coefficient , and expression  represent the number of selected lncRNA, lncRNA numbers, regression coefficient values and lncRNA expression value, respectively. Principal component analysis (PCA) was further used for dimensionality reduction, grouping visualization and model identification of the entire gene expression profiles, 403 chemokine-related genes and risk model according to the risk patterns of the risk model [25].

### Validation of the Risk Model

According to the median risk score, all samples were divided into high-risk group and low-risk group. Kaplan-Meier survival analysis was used to determine the over survival (OS) difference between the two groups. A time-dependent receiver operating characteristic (ROC) curve was plotted to detect the accuracy of the risk model. The expression of the chemokine-related lncRNAs in the model was visualized by using a heatmap. All analyses were further performed in the entire set and testing set. R package of “survivalROC”, “survival”, “survminer” and “pheatmap” were used in the validation of the risk model.

### Independent Prognostic Value of the Risk Model

The relationship between the risk model and clinicopathological characteristics was determined by using the chi-square test. Univariate analysis and multivariate analysis were used to detect the independent prognostic value of the risk model. Kaplan-Meier survival analysis was used to determine the over survival (OS) difference among patients with different clinical characteristics. The ROC curve and decision curve analysis (DCA) were performed to validate the clinical application value of the risk model. The “survival” and “regplot” R packages were utilized to construct a nomogram for the prediction of survival time in GC patients. The calibration curve was acquired to assess the accuracy of the nomogram by using “rms” package of R.

### Correlation Between the Risk Model and Immune-Related Pathway

Gene set enrichment analyses (GSEA) were performed to define the lncRNAs signatures in the KEGG. Subsequently, we obtained and evaluated the difference in immune-related pathways between the high-risk group and low-risk group through the single-sample gene set enrichment analysis (ssGSEA). In ssGSEA analysis, the R packages of “limma”, “GSVA”, “GSEABase”, “ggpubr”, “reshape2” were used.

### Evaluation of Immune Cell Infiltration

The CIBERSORT bioinformatic computational tool was used to predict the infiltration status of immune cells in tumors (Supplementary Table S9). The root mean squared error and *p*-value were counted for each sample file to improve the accuracy of the deconvolution algorithm. Only *p* < 0.05 was filtered and selected for further analysis, and the algorithm used a default signature matrix for 1000-loop computation. The “corrplot” package was used to visualize the correlation among 22 immune cells. The difference of immune infiltration cells between the high-risk and low-risk group was visualized by using R packages of “ggpubr”, “ggplot2” and “data.table”.

### The Clinical Value of the Risk Model in Immunotherapy

The expression of immune checkpoint genes between the high-risk group and low-risk group patients was evaluated by using “limma”, “reshape2”, “ggplot2” and “ggpubr” package of R. In addition, the immunotherapy score data was obtained from TCIA (Supplementary Table S10). The sensitivity of high-risk and low-risk group patients to immunotherapy was calculated to further validate the prognostic function of our risk model. Tumor Immune Dysfunction and Exclusion (TIDE) score was acquired from http://tide.dfci.harvard.edu.

### Correlation Between the Risk Model and Tumor Mutation Burden

Tumor mutation burden (TMB) data of GC was downloaded from the TCGA database (https://tcga-data.nci.nih.gov/tcga/). The correlation between the risk model and tumor mutation burden was acquired and visualized by using “ggpubr”, “reshape2” and “ggplot2” packages of R software. The “maftools” package was utilized to obtain the TMB status in the high-risk group (Supplementary Table S11) and low-risk group patients (Supplementary Table S12). Kaplan-Meier analysis was performed to determine the survival difference among patients with different statuses of TMB and risk scores.

### Human Tissue Samples Collection, RNA Isolation and Quantitative Real-Time PCR

A total of 18 pairs of GC tissues and adjacent normal tissues were collected from the First Affiliated Hospital of Chongqing Medical University (Chongqing, China). This study was approved by the Ethics Committee of the First Affiliated Hospital of Chongqing medical university. Total RNA of GC samples was isolated by using Trizol reagent according to the manufacturer’s protocol (Takara, Japan). For the qRT-PCR assay, all primers were designed and synthesized by Sangon Biotech (Sangon Biotech, Wu Han, China). cDNA was synthesized by using PrimeScript RT Reagent Kit (#RR037A, Takara, Japan). qRT-PCR was performed by using TB Green Premix Ex Taq II (Takara, #RR820A). Results were normalized using GAPDH. The information of primers was exhibited in Supplementary Table S15.

### Statistical Analysis

All data were acquired by using Perl (5.30.1) or R (version 4.1.0) software. Pearson correlation test was used for the correlation analysis. Survival analyses were performed using the Kaplan-Meier method with a log-rank test.

## Data Availability

The original contributions presented in the study are included in the article/Supplementary Material, further inquiries can be directed to the corresponding authors.

## Supplementary Material

Supplementary Figure S1 Validation of the risk model in the entire set. Prognosis of the risk model. Patients were ranked according to the risk score **(A)**. Survival analysis of the entire set **(A)**. Roc curves were plotted to assess the accuracy of the risk model in the entire set **(B)**. The survival status of the patients in the high-risk and low-risk group **(C)**. The expression of the chemokine-related lncRNA in the risk model was shown by using heatmap **(D)**. Principal component analysis (PCA) for the entire gene set **(E)**, chemokines **(F)** and lncRNAs in the risk model **(G)**. The green plots represent the patients with low risk, and the red plots represent the patients with high risk.

Supplementary Figure S2 Survival analysis was conducted in high-risk and low-risk group patients with different ages **(A)**, genders **(B)**, grades **(C)**, stages **(D)**, T stages **(E)**, different N stages **(F)** and M stages **(G)**.

Supplementary Figure S3 Survival analysis was conducted in patients without chemotherapy **(A)**. Survival analysis was conducted in patients receiving chemotherapy **(B)**. AUC value of the survival analysis in patients with chemotherapy **(C)**. 3 and 5 years DCA curves were plotted to validate the clinical application value of our risk model **(D and E)**. CIBERSORT was utilized to visualize the infiltration of 22 immune cells in the high-risk group **(F)** and low-risk group **(G)**. Prognostic function of our risk model in colorectal cancer (CRC) patients **(H)**. AUC value of our risk model in predicting OS of CRC patients **(I)**. Tumor Immune Dysfunction and Exclusion (TIDE) score was obtained to prove the immune response difference between the high-risk group and low-risk group **(J)**.

Supplementary Table S1 Clinical information of the GC patients

Supplementary Table S2 Expression of 64 Chemokines related genes in 343 GC Samples

Supplementary Table S3 Expression of 403 Chemokines related lncRNAs in 343 GC Samples

Supplementary Table S4 Prognosis-related 24 lncRNAs

Supplementary Table S5 Ten lncRNAs for the construction of the model

Supplementary Table S6 Risk pattern of all GC patients

Supplementary Table S7 Risk pattern of the training group

Supplementary Table S8 Risk pattern of the testing group

Supplementary Table S9 Immune cells infiltration status of GC patients

Supplementary Table S10 Immune therapy sensitivity data

Supplementary Table S11 Tumor mutation burden data of the high-risk group

Supplementary Table S12 Tumor mutation burden data of the low-risk group

Supplementary Table S13 The risk score of GC patients receiving chemotherapy

Supplementary Table S14 The risk score of GC patients receiving no chemotherapy

Supplementary Table S15 Quantitative real-time PCR primer sequences and R code used in our manuscript
