# Supplementary material for: Identification and Comprehensive Prognostic Analysis of a Novel Chemokine-Related lncRNA Signature and Immune Landscape in Gastric Cancer
Source: Front Cell Dev Biol. 2022 Jan 14;9:797341. doi: 10.3389/fcell.2021.797341 (PMC8795836; doi:10.3389/fcell.2021.797341)
Supplement: Supplementary file 6 [file Table15.DOC]

**Supplementary Table 15: quantitative real-time PCR primer sequences**

| Name | Primer |
| --- | --- |
| AC010719.1  MIR3142HG  BX293535.1  LINC01094  AC008770.3  AC147067.2  GAPDH | F: GTGTGTCTGTGTGTCTGTCTACCTG  R: GACACCAGAGTTCAGACCAAGCAG  F: AGTCCTTCTGTTGCCCTCCTCTG  R: AGTCTTTCACCTGCCTCTCCTCTG  F: GTGTGTCTGTGTGTCTGTCTACCTG  R: GACACCAGAGTTCAGACCAAGCAG  F: CCACCAAGTCTGCAATTCT  R: TCCTTTCTTCCTCCTGACAT  F: CACTGAACTGAGATCACGCCACTG  R: GGGTTGCTCCTTGGGCTCTTTG  F: TGGTGGGTCAGAGATCTCCTG  R: CACAGTTGCACAGACGACAGT  F: CTTTGGTATCGTGGAAGGACTC  R: GTAGAGGCAGGGATGATGTTCT |

**Supplementary Table 15: R code used in our mamuscript**

**##Construction of the model**

library("glmnet")

library("survival")

setwd("C:\\Users\\dell\\Desktop\\biotype and immune\\process 2\\2. lasso")

rt=read.table("TiranUniSigExp.txt",header=T,sep="\t",row.names=1)

x=as.matrix(rt[,c(3:ncol(rt))])

y=data.matrix(Surv(rt$futime,rt$fustat))

fit=glmnet(x, y, family = "cox", maxit = 1000)

cvfit=cv.glmnet(x, y, family="cox", maxit = 1000)

coef=coef(fit, s = cvfit$lambda.min)

index=which(coef != 0)

actCoef=coef[index]

lassoGene=row.names(coef)[index]

geneCoef=cbind(Gene=lassoGene,Coef=actCoef)

write.table(geneCoef,file="geneCoef.txt",sep="\t",quote=F,row.names=F)

**#train**

trainFinalGeneExp=rt[,lassoGene]

myFun=function(x){crossprod(as.numeric(x),actCoef)}

trainScore=apply(trainFinalGeneExp,1,myFun)

outCol=c("futime","fustat",lassoGene)

risk=as.vector(ifelse(trainScore>median(trainScore),"high","low"))

outTab=cbind(rt[,outCol],riskScore=as.vector(trainScore),risk)

write.table(cbind(id=rownames(outTab),outTab),file="Trainrisk.txt",sep="\t",quote=F,row.names=F)

**# test**

rt=read.table("Test expTime.txt",header=T,sep="\t",row.names=1)

rt[,3:ncol(rt)][rt[,3:ncol(rt)]<0]=0

testFinalGeneExp=rt[,lassoGene]

testScore=apply(testFinalGeneExp,1,myFun)

outCol=c("futime","fustat",lassoGene)

risk=as.vector(ifelse(testScore>median(trainScore),"high","low"))

outTab=cbind(rt[,outCol],riskScore=as.vector(testScore),risk)

write.table(cbind(id=rownames(outTab),outTab),file="Testrisk.txt",sep="\t",quote=F,row.names=F)

**#total**

rt=read.table("Total expTime.txt",header=T,sep="\t",row.names=1)

rt[,3:ncol(rt)][rt[,3:ncol(rt)]<0]=0

testFinalGeneExp=rt[,lassoGene]

testScore=apply(testFinalGeneExp,1,myFun)

outCol=c("futime","fustat",lassoGene)

risk=as.vector(ifelse(testScore>median(trainScore),"high","low"))

outTab=cbind(rt[,outCol],riskScore=as.vector(testScore),risk)

write.table(cbind(id=rownames(outTab),outTab),file="Totalrisk.txt",sep="\t",quote=F,row.names=F)

pdf("lambda.pdf")

plot(fit, xvar = "lambda", label = TRUE)

dev.off()

pdf("cvfit.pdf")

plot(cvfit)

abline(v=log(c(cvfit$lambda.min,cvfit$lambda.1se)),lty="dashed")

dev.off()

**##Analysis of the risk model**

library(survivalROC)

setwd("C:\\Users\\dell\\Desktop\\biotype and immune\\process\\8. trian analysis")

rt=read.table("riskTrain.txt", header=T, sep="\t", check.names=F, row.names=1)

**#ROC curve**

rocCol=c("red", "green", "blue")

aucText=c()

pdf(file="ROC.multiTime.pdf",width=5,height=5)

#1 Year

predictTime=1

par(oma=c(0.5,1,0,1),font.lab=1.5,font.axis=1.5)

roc=survivalROC(Stime=rt$futime, status=rt$fustat, marker=rt$riskScore, predict.time=predictTime, method="KM")

plot(roc$FP, roc$TP, type="l", xlim=c(0,1), ylim=c(0,1),col=rocCol[1],

xlab="False positive rate", ylab="True positive rate",

lwd = 2, cex.main=1.3, cex.lab=1.2, cex.axis=1.2, font=1.2)

aucText=c(aucText,paste0("One year"," (AUC=",sprintf("%.3f",roc$AUC),")"))

abline(0,1)

#3 Years

predictTime=3

roc=survivalROC(Stime=rt$futime, status=rt$fustat, marker=rt$riskScore, predict.time =predictTime, method="KM")

lines(roc$FP, roc$TP, type="l", xlim=c(0,1), ylim=c(0,1),col=rocCol[2],lwd = 2)

aucText=c(aucText,paste0("Three year"," (AUC=",sprintf("%.3f",roc$AUC),")"))

#5 Years

predictTime=5

roc=survivalROC(Stime=rt$futime, status=rt$fustat, marker=rt$riskScore, predict.time =predictTime, method="KM")

lines(roc$FP, roc$TP, type="l", xlim=c(0,1), ylim=c(0,1),col=rocCol[3],lwd = 2)

aucText=c(aucText,paste0("Five year"," (AUC=",sprintf("%.3f",roc$AUC),")"))

legend("bottomright", aucText,lwd=2,bty="n",col=rocCol)

dev.off()

**#Survival curve**

library(survival)

library(survminer)

bioSurvival=function(inputFile=null,outFile=null){

rt=read.table(inputFile, header=T, sep="\t")

diff=survdiff(Surv(futime, fustat) ~risk, data = rt)

pValue=1-pchisq(diff$chisq,df=1)

if(pValue<0.001){

pValue="p<0.001"

}else{

pValue=paste0("p=",sprintf("%0.3f",pValue))

}

fit <- survfit(Surv(futime, fustat) ~ risk, data = rt)

surPlot=ggsurvplot(fit,

data=rt,

conf.int=TRUE,

pval=pValue,

pval.size=6,

palette=c("red", "blue"),

legend.title="Risk",

legend.labs=c("High risk", "Low risk"),

xlab="Time(years)",

break.time.by = 1,

risk.table=TRUE,

risk.table.title="",

risk.table.height=.25)

pdf(file=outFile,onefile = FALSE,width = 5.5,height =5)

print(surPlot)

dev.off()

}

bioSurvival(inputFile="riskTrain.txt",outFile="survival.pdf")

**#Risk plot**

inputFile="riskTrain.txt"

riskScoreFile="riskScore.pdf"

survStatFile="survStat.pdf"

rt=read.table(inputFile, header=T, sep="\t", row.names=1, check.names=F)

rt=rt[order(rt$riskScore),]

riskClass=rt[,"risk"]

lowLength=length(riskClass[riskClass=="low"])

highLength=length(riskClass[riskClass=="high"])

lowMax=max(rt$riskScore[riskClass=="low"])

line=rt[,"riskScore"]

line[line>10]=10

pdf(file=riskScoreFile, width=8, height=3.5)

plot(line, type="p", pch=20,

xlab="Patients (increasing risk socre)", ylab="Risk score",

col=c(rep("skyblue",lowLength),rep("red",highLength)) )

abline(h=lowMax,v=lowLength,lty=2)

legend("topleft", c("High risk", "Low Risk"),bty="n",pch=19,col=c("red","skyblue"),cex=1.1)

dev.off()

color=as.vector(rt$fustat)

color[color==1]="red"

color[color==0]="skyblue"

pdf(file=survStatFile, width=8, height=3.5)

plot(rt$futime, pch=19,

xlab="Patients (increasing risk socre)", ylab="Survival time (years)",

col=color)

legend("topleft", c("Dead", "Alive"),bty="n",pch=19,col=c("red","skyblue"),cex=1.1)

abline(v=lowLength,lty=2)

dev.off()

**#Risk heatmap**

library(pheatmap)

bioRiskPlot=function(inputFile=null,riskScoreFile=null,survStatFile=null,heatmapFile=null){

rt=read.table(inputFile,sep="\t",header=T,row.names=1,check.names=F)

rt=rt[order(rt$riskScore),]

rt1=rt[c(3:(ncol(rt)-2))]

rt1=log2(rt1+1)

rt1=t(rt1)

annotation=data.frame(type=rt[,ncol(rt)])

rownames(annotation)=rownames(rt)

pdf(file=heatmapFile,width = 10,height = 3.5)

pheatmap(rt1,

annotation=annotation,

cluster_cols = FALSE,

fontsize_row=11,

show_colnames = F,

fontsize_col=3,

color = colorRampPalette(c("navy", "white", "firebrick3"))(50) )

dev.off()

}

bioRiskPlot(inputFile="riskTrain.txt",heatmapFile="train.heatmap.pdf")

**##Independent prognosis**

library(survival)

setwd("C:\\Users\\dell\\Desktop\\biotype and immune\\process\\11. Independent prognosis")

risk=read.table("riskTrain.txt",header=T,sep="\t",check.names=F,row.names=1)

cli=read.table("tcgaClinical.txt",sep="\t",check.names=F,header=T,row.names=1)

sameSample=intersect(row.names(cli),row.names(risk))

risk=risk[sameSample,]

cli=cli[sameSample,]

rt=cbind(futime=risk[,1],fustat=risk[,2],cli,riskScore=risk[,(ncol(risk)-1)])

**#uniCox analysis**

uniTab=data.frame()

for(i in colnames(rt[,3:ncol(rt)])){

cox <- coxph(Surv(futime, fustat) ~ rt[,i], data = rt)

coxSummary = summary(cox)

uniTab=rbind(uniTab,

cbind(id=i,

HR=coxSummary$conf.int[,"exp(coef)"],

HR.95L=coxSummary$conf.int[,"lower .95"],

HR.95H=coxSummary$conf.int[,"upper .95"],

pvalue=coxSummary$coefficients[,"Pr(>|z|)"])

)

}

write.table(uniTab,file="train.uniCox.txt",sep="\t",row.names=F,quote=F)

**# multiCox analysis**

multiCox=coxph(Surv(futime, fustat) ~ ., data = rt)

multiCoxSum=summary(multiCox)

multiTab=data.frame()

multiTab=cbind(

HR=multiCoxSum$conf.int[,"exp(coef)"],

HR.95L=multiCoxSum$conf.int[,"lower .95"],

HR.95H=multiCoxSum$conf.int[,"upper .95"],

pvalue=multiCoxSum$coefficients[,"Pr(>|z|)"])

multiTab=cbind(id=row.names(multiTab),multiTab)

write.table(multiTab,file="train.multiCox.txt",sep="\t",row.names=F,quote=F)

**#forest map**

bioForest=function(coxFile=null,forestFile=null,forestCol=null){

rt <- read.table(coxFile,header=T,sep="\t",row.names=1,check.names=F)

gene <- rownames(rt)

hr <- sprintf("%.3f",rt$"HR")

hrLow <- sprintf("%.3f",rt$"HR.95L")

hrHigh <- sprintf("%.3f",rt$"HR.95H")

Hazard.ratio <- paste0(hr,"(",hrLow,"-",hrHigh,")")

pVal <- ifelse(rt$pvalue<0.001, "<0.001", sprintf("%.3f", rt$pvalue))

pdf(file=forestFile, width = 6.3,height = 4.5)

n <- nrow(rt)

nRow <- n+1

ylim <- c(1,nRow)

layout(matrix(c(1,2),nc=2),width=c(3,2.5))

xlim = c(0,3)

par(mar=c(4,2.5,2,1))

plot(1,xlim=xlim,ylim=ylim,type="n",axes=F,xlab="",ylab="")

text.cex=0.8

text(0,n:1,gene,adj=0,cex=text.cex)

text(1.5-0.5*0.2,n:1,pVal,adj=1,cex=text.cex);text(1.5-0.5*0.2,n+1,'pvalue',cex=text.cex,font=2,adj=1)

text(3,n:1,Hazard.ratio,adj=1,cex=text.cex);text(3,n+1,'Hazard ratio',cex=text.cex,font=2,adj=1,)

par(mar=c(4,1,2,1),mgp=c(2,0.5,0))

xlim = c(0,max(as.numeric(hrLow),as.numeric(hrHigh)))

plot(1,xlim=xlim,ylim=ylim,type="n",axes=F,ylab="",xaxs="i",xlab="Hazard ratio")

arrows(as.numeric(hrLow),n:1,as.numeric(hrHigh),n:1,angle=90,code=3,length=0.05,col="darkblue",lwd=2.5)

abline(v=1,col="black",lty=2,lwd=2)

boxcolor = ifelse(as.numeric(hr) > 1, forestCol, forestCol)

points(as.numeric(hr), n:1, pch = 15, col = boxcolor, cex=1.3)

axis(1)

dev.off()

}

bioForest(coxFile="train.uniCox.txt",forestFile="train.uniForest.pdf",forestCol="green")

bioForest(coxFile="train.multiCox.txt",forestFile="train.multiForest.pdf",forestCol="red")

**##clinic ROC curve**

library(survivalROC)

riskFile="totalrisk.txt"

cliFile="tcgaClinical.txt"

setwd("C:\\Users\\dell\\Desktop\\biotype and immune\\process\\15.cliRO and DCA")

risk=read.table(riskFile, header=T, sep="\t", check.names=F, row.names=1)

risk=risk[,c("futime","fustat","riskScore")]

cli=read.table(cliFile,sep="\t",header=T,check.names=F,row.names=1)

samSample=intersect(row.names(risk), row.names(cli))

risk1=risk[samSample,,drop=F]

cli=cli[samSample,,drop=F]

rt=cbind(risk1, cli)

rocCol=rainbow(ncol(rt)-2)

aucText=c()

pdf(file="cliROC.pdf", width=5.5, height=5.5)

par(oma=c(0.5,1,0,1),font.lab=1.5,font.axis=1.5)

roc=survivalROC(Stime=risk$futime, status=risk$fustat, marker=risk$riskScore, predict.time=1, method="KM")

plot(roc$FP, roc$TP, type="l", xlim=c(0,1), ylim=c(0,1),col=rocCol[1],

xlab="False positive rate", ylab="True positive rate",

lwd = 2, cex.main=1.3, cex.lab=1.2, cex.axis=1.2, font=1.2)

aucText=c(aucText,paste0("risk score"," (AUC=",sprintf("%.3f",roc$AUC),")"))

abline(0,1)

j=1

for(i in colnames(rt[,4:ncol(rt)])){

roc=survivalROC(Stime=rt$futime, status=rt$fustat, marker = rt[,i], predict.time =1, method="KM")

j=j+1

lines(roc$FP, roc$TP, type="l", xlim=c(0,1), ylim=c(0,1),col=rocCol[j],lwd = 2)

aucText=c(aucText,paste0(i," (AUC=",sprintf("%.3f",roc$AUC),")"))

}

legend("bottomright", aucText, lwd=2, bty="n", col=rocCol)

dev.off()

**##DCA curve**

library(survival)

library(ggDCA)

riskFile="totalrisk.txt"

cliFile="tcgaclinical.txt"

risk=read.table(riskFile, header=T, sep="\t", check.names=F, row.names=1)

risk=risk[,c("futime", "fustat", "risk")]

cli=read.table(cliFile, header=T, sep="\t", check.names=F, row.names=1)

samSample=intersect(row.names(risk), row.names(cli))

risk1=risk[samSample,,drop=F]

cli=cli[samSample,,drop=F]

rt=cbind(risk1, cli)

rt[,"age"]=ifelse(rt[,"age"]>65, 1, 0) #

predictTime=5

Risk<-coxph(Surv(futime,fustat)~risk,rt)

Age<-coxph(Surv(futime,fustat)~age,rt)

Gender<-coxph(Surv(futime,fustat)~gender,rt)

Grade<-coxph(Surv(futime,fustat)~grade,rt)

Stage<-coxph(Surv(futime,fustat)~stage,rt)

T<-coxph(Surv(futime,fustat)~T,rt)

M<-coxph(Surv(futime,fustat)~M,rt)

N<-coxph(Surv(futime,fustat)~N,rt)

pdf(file="TCGADCA.pdf", width=6.5, height=5.2)

d_train=dca(Risk,Age,Gender,Grade,Stage,T,M,N, times=predictTime)

ggplot(d_train, linetype=1)

dev.off()

**##nomogram and c-index**

library(survival)

library(survminer)

library(timeROC)

library(rms)

library(regplot)

riskFile="totalrisk.txt"

cliFile="tcgaClinical.txt"

setwd("C:\\Users\\dell\\Desktop\\biotype and immune\\process\\16. normgraph")

risk=read.table(riskFile, header=T, sep="\t", check.names=F, row.names=1)

risk=risk[,c("futime", "fustat", "risk")]

cli=read.table(cliFile, header=T, sep="\t", check.names=F, row.names=1)

cli=cli[apply(cli,1,function(x)any(is.na(match('unknow',x)))),,drop=F]

cli$age=as.numeric(cli$age)

samSample=intersect(row.names(risk), row.names(cli))

risk1=risk[samSample,,drop=F]

cli=cli[samSample,,drop=F]

rt=cbind(risk1, cli)

res.cox=coxph(Surv(futime, fustat) ~ . , data = rt)

**#C-index**

sum.surv <-summary(res.cox)

c_index<-sum.surv$concordance

c_index

**#nom**

nom1=regplot(res.cox,

plots = c("density", "boxes"),

clickable=F,

title="",

points=TRUE,

droplines=TRUE,

observation=rt[1,],

rank="sd",

failtime = c(1,3,5),

prfail = T)

**#calibration curve**

time=1

f <- cph(Surv(futime, fustat) ~ age+gender+grade+stage+T+M+N+riskScore, x=T, y=T, surv=T, data=rt, time.inc=time)

cal <- calibrate(f, cmethod="KM", method="boot", u=time, m=100, B=1000)

pdf(file="TCGAcalibration1.pdf",height=4,width=6,)

plot(cal,xlab="Nomogram-Predicted Probability of 1-Year OS",ylab="Actual 1-Year OS(proportion)",col="red",sub=F)

dev.off()

time=3

f <- cph(Surv(futime, fustat) ~ age+gender+grade+stage+T+M+N+riskScore, x=T, y=T, surv=T, data=rt, time.inc=time)

cal <- calibrate(f, cmethod="KM", method="boot", u=time, m=100, B=1000)

pdf(file="TCGAcalibration3.pdf",height=4,width=6,)

plot(cal,xlab="Nomogram-Predicted Probability of 3-Year OS",ylab="Actual 3-Year OS(proportion)",col="red",sub=F)

dev.off()

time=5

f <- cph(Surv(futime, fustat) ~ age+gender+grade+stage+T+M+N+riskScore, x=T, y=T, surv=T, data=rt, time.inc=time)

cal <- calibrate(f, cmethod="KM", method="boot", u=time, m=100, B=1000)

pdf(file="TCGAcalibration5.pdf",height=4,width=6,)

plot(cal,xlab="Nomogram-Predicted Probability of 5-Year OS",ylab="Actual 5-Year OS(proportion)",col="red",sub=F)

dev.off() dev.off()

**##PCA**

library(limma)

library(scatterplot3d)

setwd("C:\\Users\\dell\\Desktop\\biotype and immune\\process\\14. PCA")

myPCA=function(input=null,output=null)

{

rt=read.table(input,sep="\t",header=T,check.names=F)

rt=as.matrix(rt)

rownames(rt)=rt[,1]

exp=rt[,2:ncol(rt)]

dimnames=list(rownames(exp),colnames(exp))

data=matrix(as.numeric(as.matrix(exp)),nrow=nrow(exp),dimnames=dimnames)

data=avereps(data)

data=data[rowMeans(data)>0.5,]

type=sapply(strsplit(colnames(data),"\\-"),"[",4)

type=sapply(strsplit(type,""),"[",1)

type=gsub("2","1",type)

data=t(data[,type==0])

rownames(data)=gsub("(.*?)\\-(.*?)\\-(.*?)\\-(.*?)\\-.*","\\1\\-\\2\\-\\3",rownames(data))

risk=read.table("totalrisk.txt",sep="\t",header=T,row.names=1)

sameSample=intersect(rownames(data),rownames(risk))

data=data[sameSample,]

risk=risk[sameSample,]

group=as.vector(risk[,"risk"])

data.class <- rownames(data)

data.pca <- prcomp(data, scale. = TRUE)

color=ifelse(group=="low",3,2)

pcaPredict=predict(data.pca)

pdf(file=output,width=5.5,height=5)

s3d=scatterplot3d(pcaPredict[,1:3], pch = 16, color=color)

legend("top", legend = c("Low risk","High risk"),pch = 16, inset = -0.2, xpd = TRUE, horiz = TRUE,col=c(3,2))

dev.off()

}

myPCA(input="symbol.txt",output="allGene.PCA.pdf")

myPCA(input="BiotypeGeneExp.txt",output="BiotypeGene.PCA.pdf")

myPCA(input="biotypeLncExp.txt",output="biotypeLnc.PCA.pdf")

risk=read.table("totalrisk.txt",sep="\t",header=T,row.names=1)

data=risk[,3:(ncol(risk)-2)]

group=as.vector(risk[,"risk"])

data.class <- rownames(data)

data.pca <- prcomp(data, scale. = TRUE)

color=ifelse(group=="low",3,2)

pcaPredict=predict(data.pca)

pdf(file="riskGene.PCA.pdf",width=5.5,height=5.5)

s3d=scatterplot3d(pcaPredict[,1:3], pch = 16, color=color)

legend("top", legend = c("Low risk","High risk"),pch = 16, inset = -0.2, xpd = TRUE, horiz = TRUE,col=c(3,2))

dev.off()

**##GSEA**

library(GSVA)

library(limma)

library(GSEABase)

inputFile="symbol.txt"

gmtFile="immune.gmt"

setwd("C:\\Users\\dell\\Desktop\\18. immune score")

rt=read.table(inputFile,sep="\t",header=T,check.names=F)

rt=as.matrix(rt)

rownames(rt)=rt[,1]

exp=rt[,2:ncol(rt)]

dimnames=list(rownames(exp),colnames(exp))

mat=matrix(as.numeric(as.matrix(exp)),nrow=nrow(exp),dimnames=dimnames)

mat=avereps(mat)

mat=mat[rowMeans(mat)>0,]

geneSet=getGmt(gmtFile, geneIdType=SymbolIdentifier())

ssgseaScore=gsva(mat, geneSet, method='ssgsea', kcdf='Gaussian', abs.ranking=TRUE)

normalize=function(x){

return((x-min(x))/(max(x)-min(x)))}

ssgseaOut=normalize(ssgseaScore)

ssgseaOut=rbind(id=colnames(ssgseaOut),ssgseaOut)

write.table(ssgseaOut,file="ssgseaOut.txt",sep="\t",quote=F,col.names=F)
