# Supplementary figures and images for: Identification and Comprehensive Prognostic Analysis of a Novel Chemokine-Related lncRNA Signature and Immune Landscape in Gastric Cancer
Source: Front Cell Dev Biol. 2022 Jan 14;9:797341. doi: 10.3389/fcell.2021.797341 (PMC8795836; doi:10.3389/fcell.2021.797341)

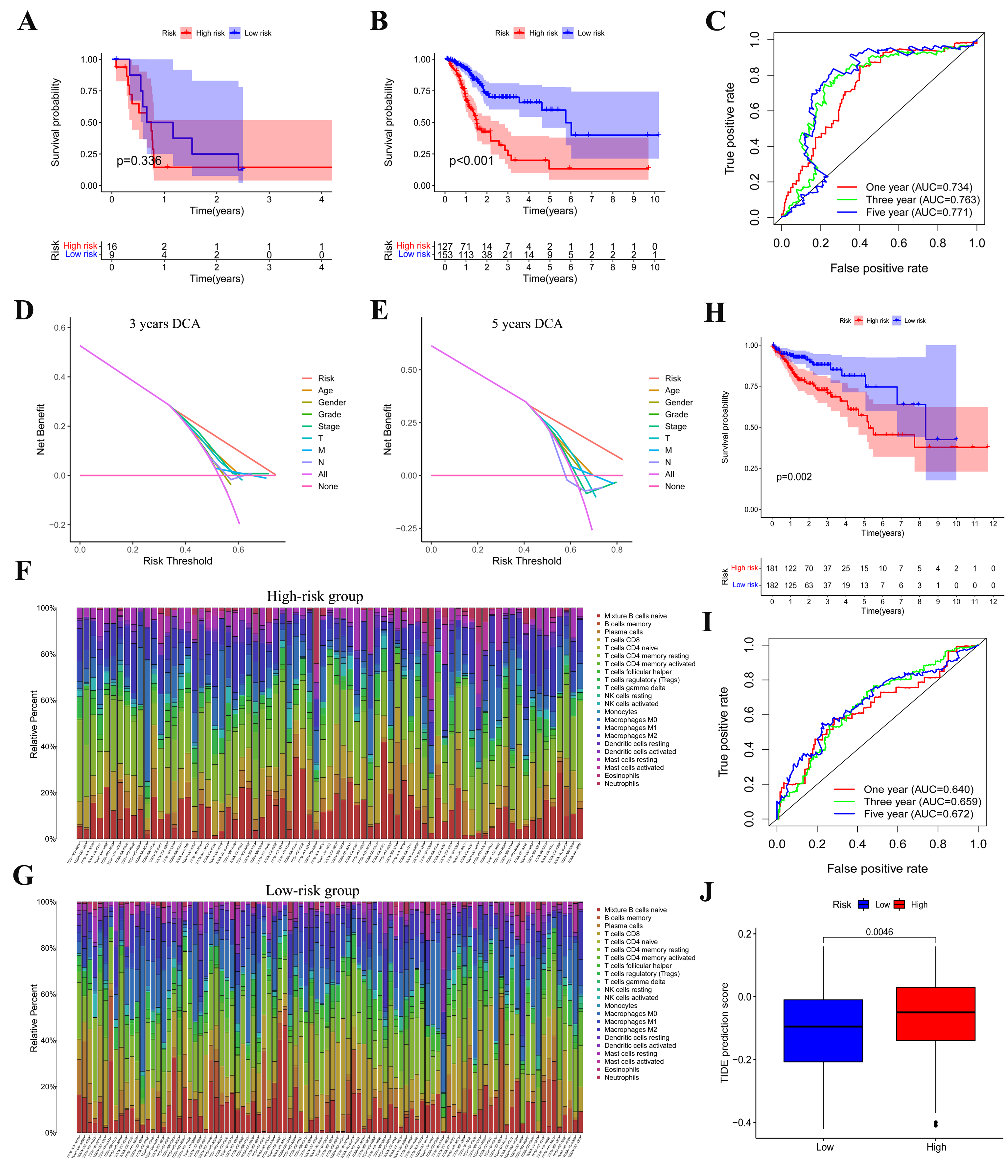

Supplement: Supplementary file 7 [file Image3.TIF]

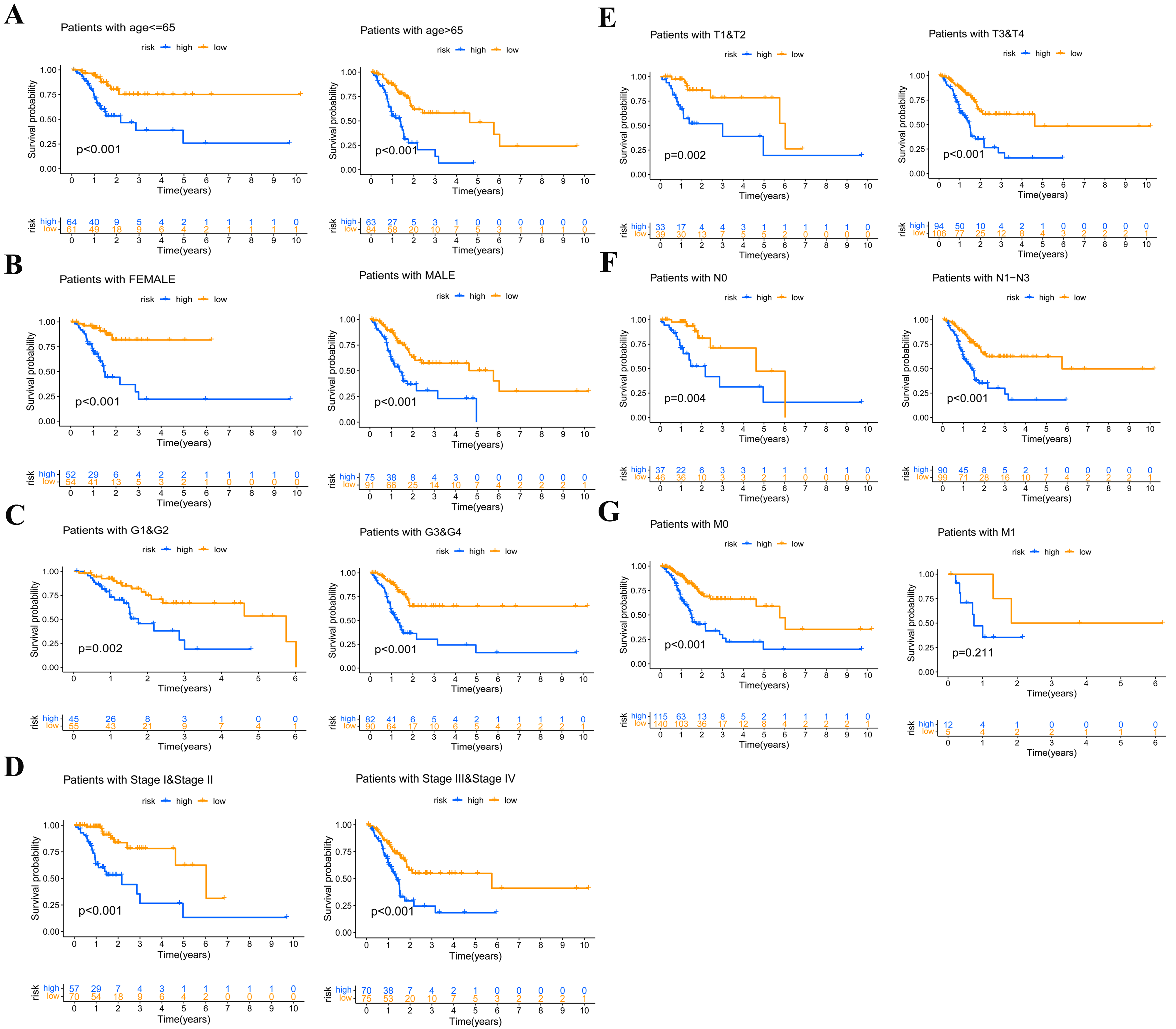

Supplement: Supplementary file 9 [file Image2.TIF]

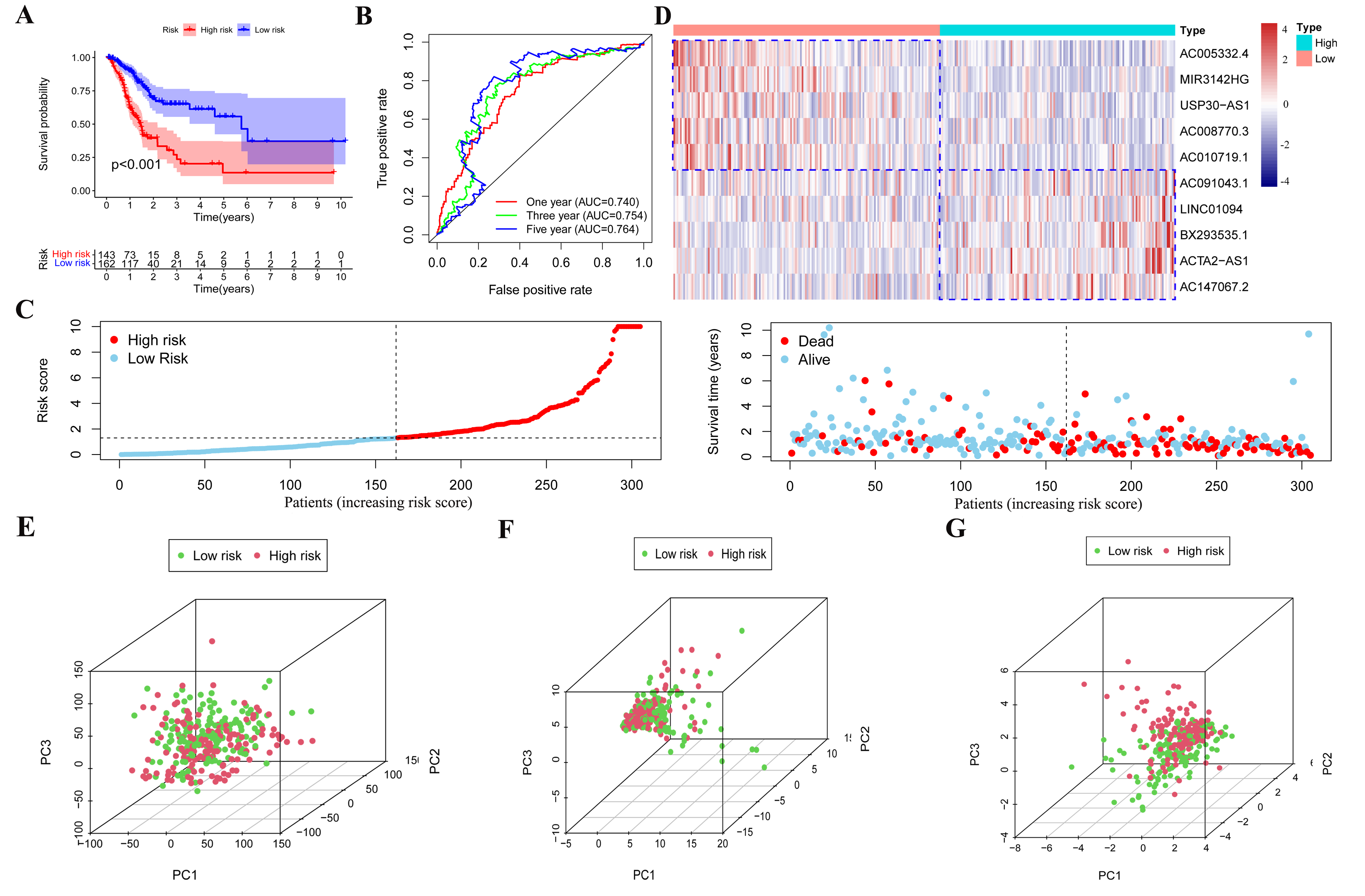

Supplement: Supplementary file 11 [file Image1.TIF]
